# *C11orf95-RELA* fusion drives aberrant gene expression through the unique epigenetic regulation for ependymoma formation

**DOI:** 10.1186/s40478-021-01135-4

**Published:** 2021-03-08

**Authors:** Tatsuya Ozawa, Syuzo Kaneko, Frank Szulzewsky, Zhiwei Qiao, Mutsumi Takadera, Yoshitaka Narita, Tadashi Kondo, Eric C. Holland, Ryuji Hamamoto, Koichi Ichimura

**Affiliations:** 1 Division of Brain Tumor Translational Research, National Cancer Center Research Institute, Chuo-ku, Tokyo, 104-0045 Japan; 2 Division of Molecular Modification and Cancer Biology, National Cancer Center Research Institute, Chuo-ku, Tokyo, 104-0045 Japan; 3 Human Biology Division, Fred Hutchinson Cancer Research Center, 1100 Fairview Avenue North, Mailstop C3-168, Seattle, WA 98109 USA; 4 Division of Rare Cancer Research, National Cancer Center Research Institute, Chuo-ku, Tokyo, 104-0045 Japan; 5 Department of Neurosurgery, Yokohama City University, Yokohama, Kanagawa 236-0027 Japan; 6 Department of Neurosurgery and Neuro-Oncology, National Cancer Center Hospital, Chuo-ku, Tokyo, 104-0045 Japan; 7 Seattle Tumor Translational Research Center, Fred Hutchinson Cancer Research Center, 1100 Fairview Avenue North, Seattle, WA 98109 USA; 8 RIKEN Center for Advanced Intelligence Project, Cancer Translational Research Team, 1-4-1 Nihonbashi, Chuo-ku, Tokyo, 103-0027 Japan

**Keywords:** Ependymoma, Fusion gene, NF-κB signaling, Transcription factor motif

## Abstract

Recurrent *C11orf95-RELA* fusions (*RELA*^*FUS*^) are the hallmark of supratentorial ependymomas. The presence of *RELA* as the fusion partner indicates a close association of aberrant NF-κB activity with tumorigenesis. However, the oncogenic role of the *C11orf95* has not been determined. Here, we performed ChIP-seq analyses to explore genomic regions bound by* RELA*^*FUS*^ and H3K27ac proteins in human 293T and mouse ependymoma cells. We then utilized published RNA-Seq data from human and mouse* RELA*^*FUS*^ tumors and identified target genes that were directly regulated by *RELA*^*FUS*^ in these tumors. Subsequent transcription factor motif analyses of* RELA*^*FUS*^ target genes detected a unique GC-rich motif recognized by the C11orf95 moiety, that is present in approximately half of* RELA*^*FUS*^ target genes. Luciferase assays confirmed that a promoter carrying this motif is sufficient to drive* RELA*^*FUS*^-dependent gene expression. Further, the* RELA*^*FUS*^ target genes were found to be overlapped with Rela target genes primarily via non-canonical NF-κB binding sites. Using a series of truncation and substitution mutants of *RELA*^*FUS*^, we also show that the activation domain in the* RELA*^*FUS*^ moiety is necessary for the regulation of gene expression of these* RELA*^*FUS*^ target genes. Lastly, we performed an anti-cancer drug screening with mouse ependymoma cells and identified potential anti-ependymoma drugs that are related to the oncogenic mechanism of *RELA*^*FUS*^. These findings suggested that *RELA*^*FUS*^ might induce ependymoma formation through oncogenic pathways orchestrated by both C11orf95 and* RELA* target genes. Thus, our study unveils a complex gene function of *RELA*^*FUS*^ as an oncogenic transcription factor in *RELA*^*FUS*^ positive ependymomas.

**Supplementary Information:**

The online version contains supplementary material available at 10.1186/s40478-021-01135-4.

## Introduction

Ependymomas are primary glial tumors that can occur in all ages and locations of the central nervous system [39]. Current therapeutic options for all tumors largely depend on maximal safe surgical resection and radiation therapy, whereas no significant survival benefit was observed for standard chemotherapy [34, 43, 68]. Recent large-scale genome sequencing studies of ependymomas have identified nine molecular subgroups associated with distinct genomic alterations, clinical behavior, age distribution, and anatomical location [44]. Supratentorial ependymomas are characterized by mutually exclusive recurrent *RELA* and *YAP1*-related gene fusions, thereby being segregated into two subgroups denoted as ST-EPN-RELA and YAP1 [44]. The ST-EPN-RELA subgroup displays a dismal prognosis, and incomplete surgical resection of these tumors is associated with increased recurrence rates and poor outcome, signifying that the development of new therapies is essential for these cases [9, 34, 44, 68]. Thus, the identification of clinically relevant subtypes and oncogenic drivers could provide the opportunity to develop specific targeted therapies for individual tumor types.

Recurrent *C11orf95-RELA* fusion (*RELA*^*FUS*^) genes were identified in a large fraction of supratentorial ependymomas [45]. *RELA*^*FUS1*^ (Type 1) and *RELA*^*FUS2*^ (Type 2), the two most frequent fusion variants are potent driver oncogenes capable of inducing human ependymoma-like tumors in mice, and therefore likely represent the tumor-initiating events in human patients [41, 45, 60]. *RELA* is a well-known master transcription factor in the NF-κB pathway, which is intimately involved in various pathophysiological processes such as inflammation and cancer [6, 69]. Upon external stimuli,* RELA* is released from IκBα-mediated cytoplasmic sequestration and translocated into the nucleus, thereby transcriptionally regulating the expression of the target genes [69]. Given that the fusion protein preferentially localizes in the nucleus, persistent activation of the NF-κB pathway is thought as the primary mechanism for the *RELA*^*FUS*^-driven ependymoma formation as bolstered by high NF-κB activity in human and mouse* RELA*^*FUS*^ tumors [41, 45]. However, dysregulation of many non-NF-κB pathways was also commonly identified in these tumors. Furthermore, expression of wild type *RELA* or activating *RELA* mutants failed to induce brain tumor formation in mice, strongly suggesting an important role of non-NF-κB pathways in the *RELA*^*FUS*^-driven ependymoma formation [41, 45].

The recent identification of active super-enhancers (SE) specific to human* RELA*^*FUS*^ ependymomas gave significant insights into the activated oncogenic pathways and potential therapeutic targets in these tumors [31]. Although a subset of these super-enhancers highlighted oncogenic driver genes and pathways associated with tumorigenesis, not all direct targets of *RELA*^*FUS*^ could be identified due to the technical limitations in accurately determining enhancer target genes [46]. Further, transcription factors generally function in a context-dependent manner [24]. Therefore, a different approach would be useful to further scrutinize genes directly regulated by *RELA*^*FUS*^. Here, to dissect the oncogenic program underlying ependymoma formation, we explored transcriptional target genes directly regulated by the* RELA*^*FUS1*^-HA protein using HA tag and H3K27ac-ChIP-seq analyses in human 293T and mouse ependymoma cells, and uncovered the complex epigenetic regulation by *RELA*^*FUS1*^. In addition, we performed an anti-cancer drug screening to validate the potential therapeutic relevance of downstream effectors driven by* RELA*^*FUS1*^ targets. Our study further deepens our understanding of the molecular functions of *RELA*^*FUS*^ in driving tumorigenesis, thus providing significant clues to identify therapeutic targets for *RELA*^*FUS*^ positive ependymomas [31].

## Materials and methods

### Generation of murine* RELA*^*FUS1*^ tumors

All animal experiments were done in accordance with protocols approved by the Institutional Animal Care and Use Committees of Fred Hutchinson Cancer Research Center (FHCRC) and followed NIH guidelines for animal welfare. The RCAS/tv-a system used in this work has been described previously [16–18, 41, 42]. Mouse* RELA*^*FUS1*^ tumors were generated with the injection of RCAS-*RELA*^*FUS1*^ or* RELA*^*FUS1*^-HA virus into newborn pups brains in *N/tv-a;Ink4a-Arf*^*−/−*^*;Pten*^*fl/fl*^ mice. The mice were sacrificed when they developed symptoms of the disease and the brain tumors were used for the generation of tumor cell lines.

### Generation of mouse neurosphere and ependymoma cell lines

Neurosphere lines were generated by mechanical dissociation from forebrains of newborn pups in *N/tv-a;Ink4a-Arf*^*−/−*^*;Pten*^*fl/fl*^ or *B/tv-a* mice and then maintained in serum-free neurosphere medium (Stem Cell Technologies) [4, 41]. Murine ependymoma cell lines were generated by mechanical dissociation from brain tumors driven by RCAS-*RELA*^*FUS1*^ (H41, H57 and H59) or RCAS-*RELA*^*FUS1*^-HA (H1203) and then maintained as adherent cells in serum-free neurosphere medium (Stem Cell Technologies) [41]

### Cell culture, transfections and infections

293T cells (ATCC: CRL-3216) and DF-1 cells (ATCC: CRL-12203) were maintained according to the manufacture’s protocol. RCAS virus was produced in DF-1 packaging cells as previously described [16–18]. Transient transfection of luciferase reporter and/or RCAS plasmids into 293T or 293T/tv-a cells was performed with X-tremeGENE 9 DNA Transfection Reagent according to the manufacture’s protocol (Merck by Roche). 293T/tv-a cell lines lentivirally expressing the tv-a-myc/6xHis were generated with standard protocol and maintained in a medium containing 1 μg/ml puromycin as previously described [42]. For the generation of 293T/tv-a cells expressing the relevant RCAS virus, cells were subjected to retroviral infection using the RCAS viral supernatant in the DF-1 cells.

### Chromatin immunoprecipitation and sequencing (ChIP-seq)

293T/tv-a cells infecting RCAS-*RELA*^*FUS1*^-HA,* RELA*^*FUS1*−S486E^-HA and mEPN cells (H41 and H1203) were fixed with 1% formaldehyde, stopped the fixation with 0.125 M glycine, and then collected ice-cold 1 × PBS(−) containing 1 mM PMSF according to the standard protocol. Nuclei preparation and chromatin digestion was performed according to manufacturer's instructions (Cell Signaling Technology, #9003) with modification [20]. Nuclei pellets were resuspended with ChIP buffer (50 mM Tris–HCl [pH 8.0], 150 mM NaCl, 1% Triton X-100, 0.5% IGEPAL CA-630, 5 mM EDTA [pH 8.0], 1 mM PMSF and Protease Inhibitor Cocktail). Samples were sonicated by using Bioruptor II (BM Equipment, BR2006A) to generate DNA fragments of ~ 200 base pairs. Antibodies (2 µg) for H3K27ac (Abcam, #4729, Lot GR3252404) or HA (Abcam, #9110, Lot GR235874-5) were added into the sheared chromatin (10 ~ 30 µg), and incubated in an ultrasonic water bath for 30 min at 4 °C. After centrifugation, supernatants were incubated with FG Beads HM Protein G (Tamagawa Seiki, TAB8848N3173) for 30 min at 4 °C. Beads were washed twice with ChIP buffer and washed with Wash buffer (50 mM Tris–HCl [pH 8.0], 300 mM NaCl, 1% Triton X-100, 0.1% SDS, 0.1% Na-deoxycholate and 5 mM EDTA pH 8.0) and LiCl buffer (50 mM Tris–HCl [pH 8.0], 250 mM LiCl, 1% Triton X-100, 0.5% Na-deoxycholate and 5 mM EDTA [pH 8.0]). Immunoprecipitated chromatin was eluted and reverse-crosslinked according to manufacturer's instructions (Cell Signaling Technology, #9003). Immunoprecipitated DNA was purified by using QIAquick PCR Purification Kit (QIAGEN, #28106). DNA libraries were prepared by using QIAseq Ultralow Input Library Kit (QIAGEN, #180492). The size determination and quantification of DNA libraries was done by qPCR (New England Biolabs, E7630) and by using Agilent 2100 Bioanalyzer. DNA libraries were sequenced on Illumina sequencers (Illumina HiSeq 3000 or NovaSeq 6000). Detailed antibody information, library preparations and Illumina sequencers in this study were described in Additional file 9: Table S1A.

### ChIP-seq data analysis

The RelA ChIP-seq datasets in murine embryonic fibroblasts after three hours of TNF stimulation were obtained from GSE132540 [37]. Sequencing reads from ChIP-seq experiments were mapped to the hg19 version of the human genome or mm9 version of the mouse genome with Bowtie (v2.2.9) and parameters–local, respectively [25]. Gene annotations were obtained from Ensemble (v.75). Duplicate reads were removed by Samtools (v1.3.1). Heatmaps to visualize enriched regions (ERs) around TSS ± 10 kb were generated with NGSplot (v2.63) [56]. The normalized ERs were visualized with the Integrative Genomics Viewer (IGV; v2.3.91) [50]. ChIP-seq ERs were called using MACS (v1.4.2) with the default parameters (*p* value cutoff; 1e-5) with the relevant input as control [70]. ChIP-seq peak statistics were summarized in Additional file 9: Table S1A. A correlation heatmap to evaluate the relationship between samples was generated by DiffBind (v2.4.8) [52]. For the differential binding analyses, we consolidated the peaks into a consensus set using a “minOverlap” of 2. The count reads were then TMM normalized implemented in DiffBind. Batch annotation of ERs was performed using ChIPpeakAnno (v3.10.2) as a Bioconductor package [73] within the statistical programming environment R (v3.4.1). Motif analyses of* C11orf95-RELA* fusion and Rela protein binding sites were performed using MEME-ChIP (v5.0.1) with sequences of ERs [30]. Overlapping peaks of ERs between different ChIP-seq experiments were obtained by using “findOverlapsOfPeaks” function under the default setting in ChIPpeakAnno. This setting counts the peaks as the minimal involved peaks in any group of connected/overlapped peaks. Super-enhancers were defined by H3K27ac peak rank order using ROSE algorithm [29, 65]. Enhancer profiles specific for human ST-EPN-RELA ependymomas and RELA-EnhancerAssociatedGene (Subgroup specific enhancers and super enhancers detected in ST-EPN-RELA ependymoma) were obtained in the previous study [31]. For the purpose of pathway enrichment analysis, gene ontology networks were generated using ClueGO (v2.5.1) [3] through Cytoscape (v3.6.1) [55]. We used the following ontologies: KEGG_20.11.2017 and REACTOME_Pathways_20.11.2017. To calculate enrichment/depletion tests, two-sided tests based on the hypergeometric distribution were performed. To correct the *p* values for multiple testing, Bonferroni step down method was used. We used min:3 max:8 GO tree interval, a minimum of 3 genes per GO term, kappa score of 0.4.

Lists of the target gene were generated by removing duplicate gene annotations from the peak list obtained at TSS ± 10 kb (Additional file 9: Table S1E). A list of ‘previously reported-NFkB target genes’ was previously described [12, 41]. For comparison of our mouse gene list to the human, all mouse gene symbols were converted to humans using the MGI homology map with the BioMart browser in the Ensembl database. The normalized ChIP-seq tracks were visualized on the IGV genome browser (v2.3.91).* RELA*^*FUS1*^-HA,* RELA*^*FUS1−S486E*^-HA, Rela, H3K27ac and input peaks are shown with the same scale in each figure (Fig. 5b, c, i, j, Additional file 5: S4B, E, F and Additional file 7: S6D). 5′-BGKGGCCCCBG-3′ (B = C or G or T, K = G or T) and 5′-GGGRNWYYCC-3′ (R = A or G, N = any base, W = A or T, and Y = C or T) sequences were used to find a genomic position of the 293T-*RELA*^*FUS1*^-MEME-2 and canonical NF-κB consensus motif, respectively. The position of the 293T-RELA^FUS1^-MEME-2 and κB site is shown as a blue vertical bar on positive (+) and negative (−) DNA strands. Transcriptional start sites (TSSs) were analyzed using the DBTSS; Data Base of Transcriptional Start Sites online tool [59]. Representative images in two technical replicates were shown in the figures.

### RNA-seq datasets and gene expression analysis

For gene expression analyses, RNA-seq datasets of human ependymomas and mouse brain tissues were obtained from the Pediatric Cancer Genome Project (PCGP, EGAS00001000254) and the Gene Expression Omnibus (GSE93765), respectively [7, 41, 45]. The human ependymoma samples were analyzed between *RELA*^*FUS*^ positive ST-EPNs and all negative ependymomas including *RELA*^*FUS*^ negative ST-EPNs and PF-EPNs. The aligned reads were counted for gene associations against the Ensemble genes database with featureCounts (v1.5.0) [27]. Transcriptomic signature genes of ST-EPN in single-cell RNA-seq analysis were obtained in the previous publication and used for an overlapping analysis with* RELA*^*FUS1*^ target genes [13]. Microarray gene expression data of the human ST-EPN-RELA and YAP1 subgroup was obtained from publicly available data (GSE64415) and used for pathway enrichment analysis [44]. Differential expression analyses were performed using the R/Bioconductor package edgeR (v3.18.1) [51]. Statistical analyses were performed using GraphPad Prism (v7.0.0).

### Vector constructs

All vectors and sgRNA target sequences used in this study are listed in Additional file 14: Table S6A. The DNA fragments for the *RELA*^*FUS1*^ and *C11orf95* mutant vector construction were PCR-amplified on the relevant DNA template to obtain appropriate restriction sites and then inserted in RCAS vector.

The luciferase reporter vectors for the* RELA*^*FUS1*^-motifs were generated by insertion of the synthetic oligonucleotide for the relevant motif sequence as between Kpn I and EcoRV sites in the pNL3.2 vector containing the minimal promoter (Promega). The C11orf95 5′-upstream luciferase reporter construct was generated by PCR-amplifying the human *C11orf95* gene sequence from genomic DNA of 293T cells. The PCR fragment was then subcloned into pGEM-T easy vector (Promega) to obtain appropriate restriction sites and subsequently inserted into the pNL3.2 (minimal promoter) vector (Promega). For RCAS-C11orf95-NLS-VP64-HA (CNVP-HA) and lentiCRISPRv2-dCas9-sgGFP1 vector construction, SV40-NLS-VP64-HA and sgGFP target sequences as previously described, were synthesized and inserted in the relevant vector, respectively [47, 54].

pLJM1-EGFP was a gift from David Sabatini (Addgene plasmid #19319; http://n2t.net/addgene:19319; RRID: Addgene_19319) [53]. psPAX2 was a gift from Didier Trono (Addgene plasmid #12260; http://n2t.net/addgene:12260; RRID: Addgene_12260). pMD2.G was a gift from Didier Trono (Addgene plasmid #12259; http://n2t.net/addgene:12259; RRID: Addgene_12259). lentiCRISPR v2-dCas9 was a gift from Thomas Gilmore (Addgene plasmid #112233; http://n2t.net/addgene:112233; RRID: Addgene 112233).

### Western blot analysis

Cells were cultured, lysed, and processed for western blotting as previously described [41]. Antibodies were listed in Additional file 14: Table S6C. All western blot analyses were performed at least twice and successfully repeated in the experiments. The representative images were shown in the figures.

For immunoblot and qPCR analyses in 293T cells as shown in Fig. 3j–l, 5g and Additional file 5: Fig. S4C, RCAS vectors were transiently transfected with the indicated plasmid concentration to adjust the protein expression level between samples. After 48 h of the transfection, cells were collected and split for RNA and protein extractions. Then, the cell lysates were subjected to immunoblot analysis with the indicated antibodies. RNAs were used for subsequent qPCR analysis.

### Quantitative PCR (qPCR) analysis

Total RNAs were extracted from mEPN cells or 293T cells using the miRNeasy or RNeasy Mini kit (QIAGEN) and were used to synthesize cDNA by using the SuperScript IV VILO Master Mix (Thermo Fisher Scientific) according to the manufacturer’s protocol. SYBR Green real-time PCR was performed using the relevant gene-specific primer sets, PowerUp SYBR Green Master Mix (2X) (ThermoFisher SCIENTIFIC), and Fast run protocol from Applied Biosystems in a QuantStudio 6 Flex Real-Time PCR System. The ΔΔCt method was used to calculate the relative gene expression normalized to the reference gene (*RPS18 or Rps18*). Each biological replicate in the PCR reaction was assayed in four technical replicates. Data (mean ± SD) are displayed as the relative ratio to the relevant control sample (e.g. GFP cells). Circles in the figure indicate relative mean values of each biological replicate. Analysis was done using paired two-tailed t-test. *p < 0.05; **p < 0.01; ***p < 0.001; ****p < 0.0001. All primers used in this study are listed in Additional file 14: Table S6B [64].

### Luciferase reporter assay

293T/tv-a cells retrovirally infecting the relevant RCAS virus were seeded at a density of 7.5 × 10^4^ cells in a 24 well format in triplicates the day prior to transfection. Cells were then co-transfected pGL4.53[luc2/PGK] (control vector) and pNL3.2 (test vector) with 1:9 ratio (total 0.2 μg/well) using X-treamGENE9 transfection reagent (Roche) in 500 μL/well of culture medium. After 24 h of the transfection, cells were lysed with the Passive Lysis Buffer (Promega E1941) (100 μL/well) and the lysates of 80 μL/well were transferred in white 96 well plates, followed by analyzed for luciferase activity using the Nano-Glo Dual-Luciferase Reporter Assay System (Promega N1630) on a GloMax Explorer luminometer (Promega) according to manufacturer’s protocol.

For analysis in transient expression of RCAS vectors as shown in Fig. 3g, h, 5h, 293T cells were seeded at a density of 5 × 10^5^ cells in a 6 well format the day prior to transfection. Cells were then co-transfected pGL4.53[luc2/PGK] (control vector), pNL3.2 (test vector) and RCAS vector with 1:9:10 ratio (total 2 μg) using X-treamGENE9 transfection reagent (Roche) in 2 mL/well of culture medium. After 24 h of the transfection, cells were lysed with the Passive Lysis Buffer of 500 μL/well and the lysates of 80 μL/well were transferred in white 96 well plates in triplicate, followed by analyzed for luciferase activity as well.

Relative luciferase activity was calculated as the ratio of NanoLuc normalized to Firefly luciferase and GFP control cells. The box plots in all luciferase reporter assays extend from the 25th to 75th percentiles. Whiskers of all box plots extend to the most extreme data point. Circles in the box plots indicate relative mean values of each biological replicate. Analysis was done using repeated measures (RM) one-way ANOVA or paired two-tailed t-test using Graph-Pad Prism 8 software, and a value of *p* < 0.05 was considered significant. *p < 0.05; **p < 0.01; ***p < 0.001; ****p < 0.0001.

#### Immunofluorescence

For analysis of subcellular localization of C11orf95-HA, RELA-HA and* RELA*^*FUS1*^-HA proteins, DF-1 cells infecting the relevant RCAS viruses grown on Lab-Tek Chamber Slide (Nalge Nunc International, Rochester, NY) were fixed with 4% paraformaldehyde and were then permeabilized with 0.2% Triton X-100 in PBS. Subsequently, cells were immunostained with an anti-HA tag (Roche, 11867423001) antibody, followed by Alexa Fluor 488 rabbit anti-rat IgG secondary antibody (Invitrogen #A21210). The analysis was performed by immunofluorescent microscopy (Leica DMI6000 microscope, FW4000 software). GFP fusion proteins were observed using fluorescent microscopy (OLYMPUS CKX53 microscope and cellSens Standard software).

### Anti-cancer drug screening

H41- and H1203-mEPN cells (5,000/well) were seeded on a 384-well culture plate in mouse neurosphere medium in duplicate (Stem Cell Technologies) and cultured overnight at 37 °C with 5% CO_2_ (day 0). Ten micromolar of 164 FDA-approved anti-cancer drugs and 15 selected potential NF-κB inhibitors (final 0.1% dimethyl sulfoxide, DMSO) was then added to the cells using the Bravo Automated liquid handling platform (Agilent technologies) (day 1), and cell viability was assessed with a CCK-8 kit (Cell Counting Kit-8, Dojindo, Kumamoto, Japan) after 72 h of incubation (day1 to day 4) (Additional file 13: Table S5A, B). The mean cell viability (% of control) was calculated (n = 2) as follows: (A sample-A blank)/(A 0.1% DMSO-treated control-A blank) (where, A = Absorbance at 450 nm) and experiments were repeated twice. Two Sorafenib Tosylate (SPL30 and SPL180) and Vorinostat (SPL70 and SPL179) from the different suppliers were tested in this screening because of redundancy between two drug libraries.

To determine the cell sensitivity to these drugs, anti-cancer drugs serially diluted in mouse neurosphere medium were added to the mEPN cells in duplicate and cultured for 72 h. Cell viability was then assessed by a CCK-8 kit as well. IC50 values were calculated by drawing four-parameter curve fitting using GraphPad Prism (version 7, GraphPad Software).

### Protein domain illustrations

Illustrations of protein domain as shown in Fig. 4a, Additional file 2: Fig. S1D, Additional file 6: Fig. S5B, G and Additional file 8: Fig. S6F were generated using the IBS software (illustrator of biological sequences) [28]. In Fig. 4a, Additional file 2: Fig. S1D and Additional file 8: Fig. S6F, Blue and red boxes represent portions of C11orf95 (UniProtKB—C9JLR9) and RELA (UniProtKB—Q04206) coding sequences, respectively. ZF, zinc finger (SPIN-DOC-like, zinc-finger); RHD, Rel homology domain; TAD, transactivation domain; black boxes in the C-terminus, HA-tag; NLS, nuclear localization signal. NLS prediction in C11orf95 protein was done using the cNLS mapper online tool (http://nls-mapper.iab.keio.ac.jp/cgi-bin/NLS_Mapper_form.cgi) [22]. The amino acid position of three predicted bipartite NLSs (210–239, 254–286 and 456–487) in C11orf95 are shown as black bars and the yellow box (cut-off score = 4.0) in Fig. 4a.

### Statistical analysis

Statistical analyses in this study were performed using GraphPad Prism 7, 8, or R software and described with the significance values and sample size in the respective figure legends, corresponding results sections, or methods section in detail.

### Materials availability

All cell lines, plasmids and other reagents generated in this study are available from the corresponding authors with a completed Materials Transfer Agreement if there is potential for commercial application.

## Results

### HA tag ChIP-seq analyses identified unique genomic binding sites of* RELA*^*FUS1*^

**Fig. 1:**
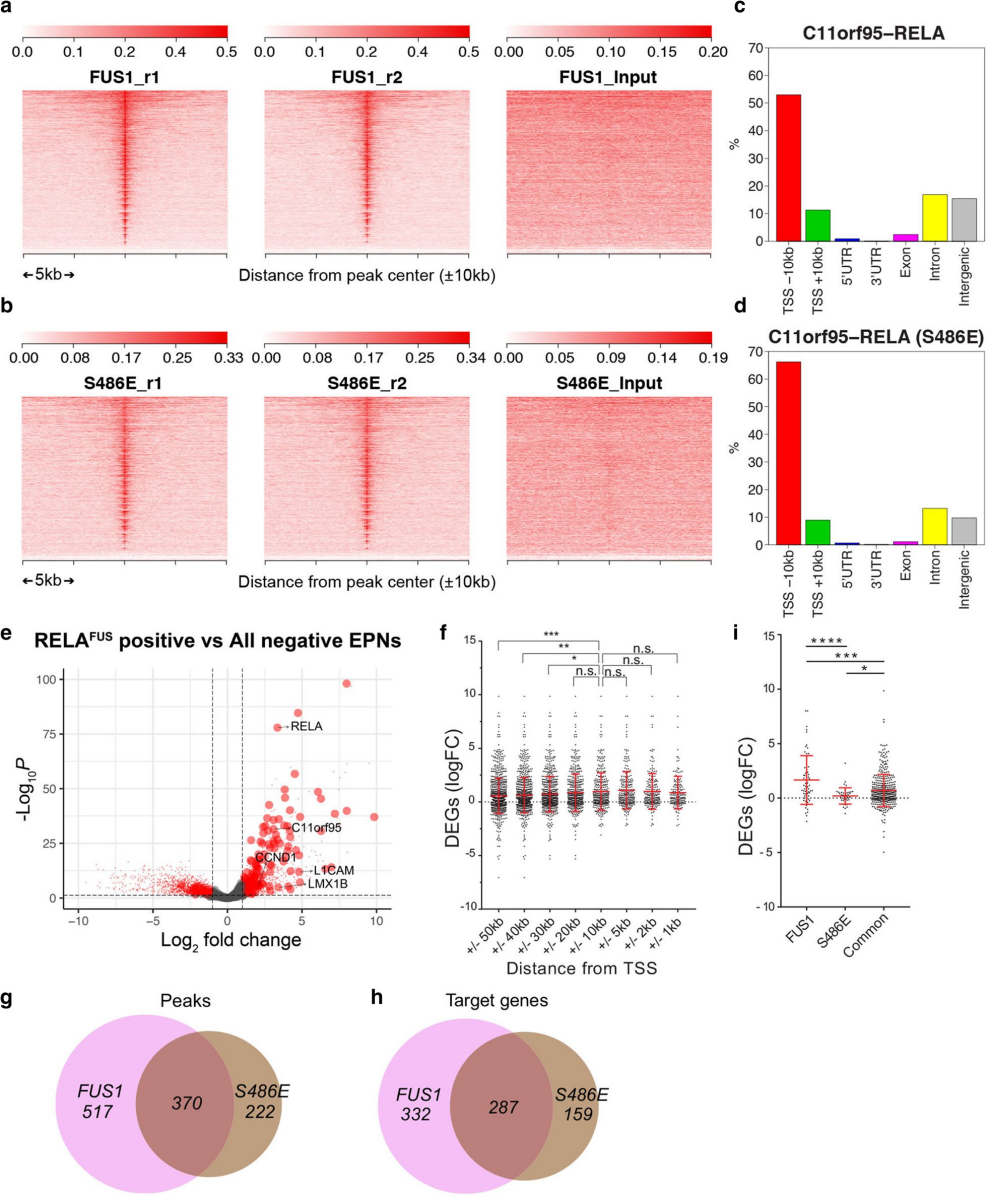
HA tag ChIP-seq analyses identified unique genomic binding sites of* RELA*^*FUS1*^. **a**, **b** Genome-wide* RELA*^*FUS1*^-HA (**a**) and* RELA*^*FUS1−S486E*^-HA (**b**) binding demonstrated by HA ChIP-seq analyses in 293T/tv-a cells. Heatmaps represent HA ChIP enrichments at the regions (TSS ± 10 kb) ranked by the sum of the enrichment values. Heatmaps for two biological replicates are shown individually. Input controls are also shown. **c**, **d** Bar graphs display the genomic distribution of the* RELA*^*FUS1*^-HA (**c**) and RELA^*FUS1−S486E*^-HA (**d**) peaks in 293T/tv-a cells. **e** Volcano plot illustrating differences in gene expression between human *RELA*^*FUS*^ positive and all negative ependymomas (DEGs: false discovery rate (FDR) < 0.05, n = 5507). Differences in Log2 fold change in gene expression values are plotted on the x-axis. Adjusted *p* values calculated using the Benjamin-Hochberg method are plotted on the y-axis.* RELA*^*FUS1*^ target genes annotated within the TSS ± 10 kb are shown as large circles. *LMX1B*, *C11orf95, RELA, CCND1* and *L1CAM* genes are indicated by the arrows. **f** Scatter plots showing the mRNA expression of direct* RELA*^*FUS1*^ target genes in the differentially expressed genes (DEGs) (logFC) between human *RELA*^*FUS*^ positive and all negative ependymomas. Genes are grouped based on the distance of the* RELA*^*FUS1*^ binding to location to the TSS of the gene (FDR < 0.05, ± 50 kb- ± 1 kb; n = 870, 769, 645, 501, 350, 261, 214 and 179). The expression level of DEGs bound by* RELA*^*FUS1*^ within the TSS ± 10 kb were compared to those bound by* RELA*^*FUS1*^ at other distances as indicated in the x-axis. **g**, **h** Venn diagram showing the number of the overlapping between the* RELA*^*FUS1*^ and* RELA*^*FUS1−S486E*^ peaks (**g**) and target genes (**h**) identified within the TSS ± 10 kb in 293T/tv-a cells. **i** Scatter plots showing the mRNA expression of target genes differentially bound by* RELA*^*FUS1*^ and/or RELA^*FUS1−S486E*^ in the DEGs (logFC) between human *RELA*^*FUS*^ positive and all negative ependymomas. The target genes of FUS1 (DBPs for* RELA*^*FUS1*^: logFC^DBPs^ > 1, FDR < 0.05, n = 64), S486E (DBPs for RELA^*FUS1−S486E*^: logFC^DBPs^ > 1, FDR < 0.05, n = 42) and common (No diffenrential peaks between* RELA*^*FUS1*^ and RELA^*FUS1−S486E*^: |logFC|^DBPs^ < 1, n = 404) are shown in the x-axis. DBPs: Differential binding peaks. Statistical differences were assessed with a Mann–Whitney U-test. *p < 0.05; **p < 0.01; ***p < 0.001; ****p < 0.0001. n.s.: not significant

Accumulating evidence suggests that—in addition to known NF-κB targets—also non-NF-κB target genes contribute to the tumorigenesis of *RELA*^*FUS*^ [31, 35, 41, 45]. Therefore, to identify direct transcriptional target genes of *RELA*^*FUS*^ and to clarify the mechanisms of how *RELA*^*FUS*^ causes tumor formation, we initially performed an HA-tag protein immunoprecipitation and sequencing (HA ChIP-seq) analysis on 293T/tv-a cells retrovirally infected with either RCAS-*RELA*^*FUS1*^-HA or* RELA*^*FUS1−S486E*^-HA (A serine-to-glutamine substitution at Ser-486 of* RELA*^*FUS1*^ corresponding to Ser-276 in the Rel homology domain of RELA, which has been previously shown to severely impair the tumor-forming capacity of *RELA*^*FUS1*^) (Additional file 2: Fig. S1A–D; Additional file 9: Table S1A) [41]. HA ChIP-seq analyses identified a large number of significant* RELA*^FUS1^ DNA-binding sites throughout the genome (Fig. 1a, b, Additional file 2: Fig. S1E; Additional file 9: Table S1B, C). Interestingly,* RELA*^*FUS1*^ and* RELA*^*FUS1−S486E*^ presented an overall similar DNA-binding pattern (Fig. 1a, b). However,* RELA*^*FUS1*^ peaks showed somewhat higher enrichment in intronic and intergenic regions but a lower enrichment in proximal promoter regions compared to* RELA*^*FUS1−S486E*^ (Fig. 1c, d), signifying an existence of DNA regulatory elements specific for* RELA*^*FUS1*^ as previously described [31].

We then determined which transcriptional target genes of *RELA*^*FUS1*^ might be involved in *RELA*^*FUS1*^-driven ependymoma formation. We used publicly available expression data of human ependymomas [45] and detected significantly higher expression of* RELA*^*FUS1*^ target genes (identified by our ChIP-seq analysis) in human *RELA*^*FUS*^ positive ependymomas compared to *RELA*^*FUS*^ negative ependymomas (Fig. 1e). Further, up-regulation of many* RELA*^*FUS1*^ target genes was also observed in a second ependymoma dataset (Additional file 2: Fig. S1F) [44]. We focused our subsequent analysis on genes bound by* RELA*^*FUS1*^ at ± 10 kb of the transcription start sites (TSSs), since these genes were significantly up-regulated compared to genes bound at ±30, 40, or 50 kb of the TSSs (Fig. 1f). We observed significant peaks of both* RELA*^*FUS1*^ and RELA^*FUS1−S486E*^ in these loci and identified 619 (in 887 peaks) and 446 (in 592 peaks) direct target genes of* RELA*^*FUS1*^ and RELA^*FUS1−S486E*^, respectively (Fig. 1g, h; Additional file 9: Table S1B–E). Interestingly, more than half of the RELA^*FUS1−S486E*^ target genes (64%; 287 out of 446 RELA^*FUS1−S486E*^ target genes) still overlapped with the* RELA*^*FUS1*^ target genes (46%; 287 out of 619* RELA*^*FUS1*^ target genes) (Fig. 1h). Notably, when comparing the expression of genes that were occupied by either only* RELA*^*FUS1*^ or only* RELA*^*FUS1−S486E*^ (at TSS ± 10 kb) in human *RELA*^*FUS*^ positive versus negative ependymomas, we observed a significantly lower up-regulation of RELA^*FUS1−S486E*^ target genes (Fig. 1i), signifying that the transcriptional activity of the mutant form might be somewhat impaired, thus likely explaining the lack of the tumor-forming potential [41]. These results suggest that *RELA*^*FUS1*^ might function as a transcription factor and induce aberrant gene expression for ependymoma formation.

### Most* RELA*^*FUS1*^ target genes are actively transcribed

**Fig. 2:**
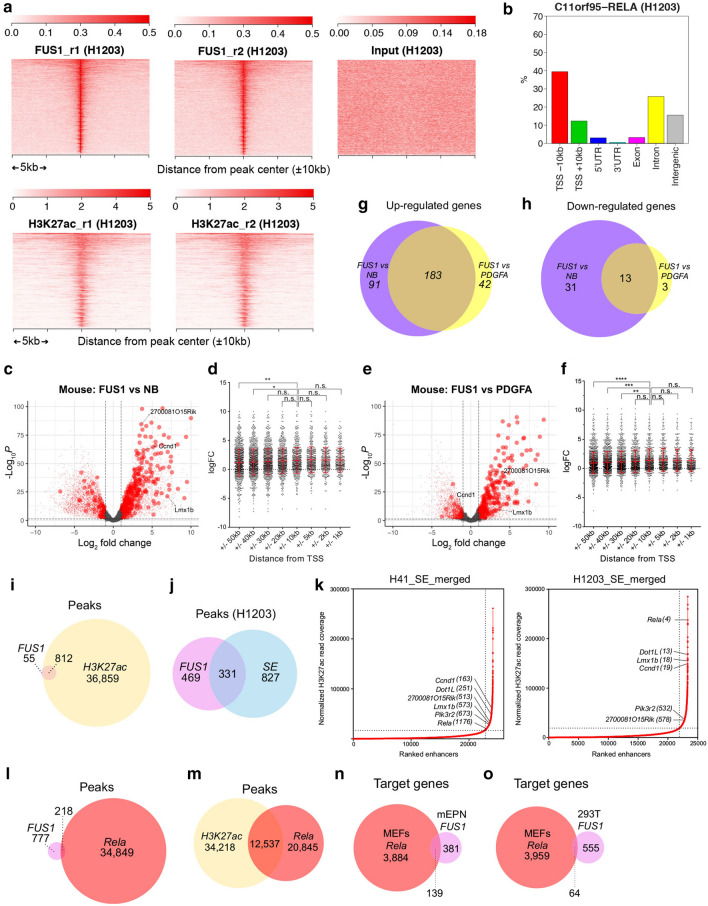
Most* RELA*^*FUS1*^ target genes are actively transcribed. **a** Genome-wide* RELA*^*FUS1*^-HA binding or H3K27ac marks in mEPN (H1203) cells. The enrichment of H3K27ac within the* RELA*^*FUS1*^-HA binding region is shown in heatmaps. **b** Bar graphs display the genomic distribution of the* RELA*^*FUS1*^ peaks in H1203 cells. **c**, **e** Volcano plots illustrating differences in gene expression between RCAS-*RELA*^*FUS1*^-driven ependymomas (FUS1) and mouse normal brains (NB) (**c**) or RCAS-*PDGFA*-driven glioblastomas (PDGFA) (**e**) (FDR < 0.05, n = 11,574 (**c**), n = 8,059 (**e**)). Differences in Log2 fold change in gene expression values are plotted on the x-axis. Adjusted *p* values calculated using the Benjamin-Hochberg method are plotted on the y-axis.* RELA*^*FUS1*^ target genes annotated within the TSS ± 10 kb in H1203 cells are shown as large circles. *2700081O15Rik*,* Ccnd1 and Lmx1b* genes are indicated by the arrows. **d**, **f** Scatter plots showing the mRNA expression of direct* RELA*^*FUS1*^ target genes in the DEGs (logFC) between *RELA*^*FUS1*^-driven ependymomas and normal brains (**d**) or *PDGFA*-driven glioblastomas (**f**). Genes are grouped based on the distance of the* RELA*^*FUS1*^ binding to location to the TSS of the gene (FDR < 0.05, ± 50 kb- ± 1 kb; n = 1,083, 943, 826, 668, 511, 412, 342 and 295). The expression level of DEGs bound by* RELA*^*FUS1*^ within the TSS ± 10 kb were compared to those bound by* RELA*^*FUS1*^ at other distances as indicated in the x-axis. **g**, **h** Venn diagram showing the number of the overlapping of up- and down-regulated* RELA*^*FUS1*^ target genes (within TSS ± 10 kb) in *RELA*^*FUS1*^-driven ependymomas relative to normal brains or *PDGFA*-driven glioblastomas. **i** Venn diagram showing the number of the overlapping of H3K27ac peaks in H41 plus H1203 cells and* RELA*^*FUS1*^ peaks in H1203 cells. **j** Venn diagram showing the number of the overlapping of* RELA*^*FUS1*^ peaks and super-enhancers (SEs) identified in H1203 cells. **k** Inflection plots representing SEs in H41 and H1203 cells. Representative genes are shown along with their rankings. **l**, **m** Venn diagrams showing the number of the overlapping of Rela peaks in MEFs after 3 h of TNF stimulation (see Methods) and* RELA*^*FUS1*^ peaks in H1203 cells (**l**) or H3K27ac peaks within the entire genome (**m**) in H41 and H1203 cells. **n**, **o** Venn diagrams showing the number of the overlapping of Rela target genes in MEFs and* RELA*^*FUS1*^ target genes in H1203 (**n**) or 293T (**o**) cells. Statistical tests are as described in Fig. 1

To further characterize* RELA*^*FUS1*^ target genes, we also investigated the transcriptional profile of *RELA*^*FUS1*^ in mouse ependymoma (mEPN) cells derived from the RCAS-RELA^FUS1^-HA-driven ependymoma (H1203 cells) (Additional file 3: Fig. S2A; Additional file 9: Table S1A) [41]. HA ChIP-seq analysis of the mEPN cells successfully identified a large number of the* RELA*^*FUS1*^ binding sites throughout the entire genome (Fig. 2a; Additional file 10: Table S2A). We observed a higher frequency of* RELA*^*FUS1*^ peaks in intronic and a lower frequency in proximal promoter regions in mEPN cells, compared to 293T-RELA^FUS1^ cells (Figs. 1c, 2b). We then examined the expression levels of* RELA*^*FUS1*^ target genes in RNA-seq data of normal mouse brains, *PDGFA*-driven gliomas, and *RELA*^*FUS1*^-driven ependymomas (Fig. 2c–f) [41]. We observed that many* RELA*^*FUS1*^ target genes were significantly up-regulated in *RELA*^*FUS1*^-driven ependymomas compared to both normal brains (Fig. 2c, d) and *PDGFA*-driven gliomas (Fig. 2e, f). We again observed that* RELA*^*FUS1*^ target genes bound within ± 10 kb of the TSS were significantly up-regulated (Fig. 2d, f) and thus focused our subsequent analysis on these genes. We identified 520* RELA*^*FUS1*^ target genes from 649* RELA*^*FUS1*^ peaks in the mEPN cells (Additional file 9: Table S1E, Additional file 10: Table 2A). We observed that a significant portion of these genes was commonly up- or down-regulated in *RELA*^*FUS1*^-driven mouse ependymomas compared to normal brains or *PDGFA*-driven gliomas, indicating the creation of a* RELA*^*FUS1*^-specific transcriptional network (Fig. 2g, h). Of note, dysregulation of PDGF signaling in human and mouse* RELA*^*FUS*^ tumors has been previously shown [41, 44, 45]. However, it is noteworthy that the* RELA*^*FUS*^-specific transcriptional program was observed even when compared to the *PDGF*-driven mouse glioma samples in our analysis, thus implying that *RELA*^*FUS*^ likely induces tumor formation by co-activating several oncogenic pathways driven by the activation of several* RELA*^*FUS*^ target genes in addition to PDGF signaling.

Subsequently, to examine whether these* RELA*^*FUS1*^ target genes were actively transcribed, we performed H3K27ac ChIP-seq (a histone mark of active chromatin) with two mEPN (H41 and H1203) cells and investigated actively transcribed regions, including promoters and enhancers (Fig. 2a, Additional file 3: Fig. S2A, B; Additional file 9: Table S1A, Additional file 10: Table S2B, C). We identified 36,859 peaks that were present in both mEPN cells (Fig. 2i, Additional file 3: Fig. S2C). Interestingly, most of the* RELA*^*FUS1*^ peaks in the TSS ± 10 kb region overlapped with H3K27ac peaks (94%; 812 out of 867* RELA*^*FUS1*^ peaks, *p* = 9.4 × 10^–271^) (Fig. 2i). Furthermore, 41% of* RELA*^*FUS1*^ peaks overlapped with super-enhancers (SEs) identified by an exceptionally high degree of enrichment of H3K27ac peak (Fig. 2j, k, Additional file 3: Fig. S2D; Additional file 10: Table S2D–F) [48, 65]. We noticed that some* RELA*^*FUS1*^ peaks (TSS ± 10 kb) overlapped with SE regions that were annotated to well-known cancer-associated genes such as *CCND1* (a representative NF-κB target gene) [14, 15]*, PIK3R2* (proto-oncogene)*, DOT1L* (histone modifier gene) in addition to *RELA* and *2700081O15Rik* (mouse homolog of *C11orf95*) (Fig. 2k; Additional file 10: Table S2F). Further, we also found that enhancer- and SE-annotated genes identified in mouse ependymoma cells and human* RELA*^*FUS*^ tumors were significantly overlapping (Additional file 3: Fig. S2E, F), thus supporting a close association of our analysis with human ependymomas [31].

To further dissect the molecular mechanism of *RELA*^*FUS*^-driven ependymoma formation, we examined the implication of RELA target genes in the* RELA*^*FUS1*^ transcription network using publicly available Rela ChIP-seq data in murine embryonic fibroblasts (MEFs) after TNF stimulation [37]. We found that approximately 22% of* RELA*^*FUS1*^ peaks in mEPN cells overlapped with Rela peaks in MEFs (Fig. 2l) as also supported by a significant overlap between the H3K27ac peaks in mEPN cells and Rela peaks in MEFs (Fig. 2m). More specifically, approximately 27 and 10% of* RELA*^*FUS1*^ target genes in mEPN and 293T-RELA^FUS1^ cells overlapped with the Rela target genes in MEFs, respectively, indicating a critical implication of RELA target genes in *RELA*^*FUS1*^-driven ependymoma formation (Fig. 2n, o). Interestingly, 27% of the previously reported-NF-κB target genes (n = 366) were present in the Rela-target genes (Additional file 3: Fig. S2G) [12, 41]. By contrast, only 1.1 and 4.4% of these NF-κB target genes were identified in the* RELA*^*FUS1*^ target genes in 293T-RELA^FUS1^ and H1203 cells, respectively, highlighting the importance of yet unknown- or non-NF-κB target genes in the *RELA*^*FUS*^-driven ependymoma formation (Additional file 3: Fig. S2H, I).

Recent single-cell RNA sequencing analyses of ependymomas identified diverse neoplastic subpopulations characterized by specific transcriptomic signatures [11, 13]. We also examined an association between the* RELA*^*FUS1*^ target genes and the single-cell transcriptomic signature genes of ST-ependymomas [13] and observed a significant overlap between “ST-RELA-variable” signature genes and* RELA*^*FUS1*^ target genes in both 293T-RELA^FUS1^ and mEPN cells, likely implying an important role of this subpopulation on tumorigenesis (Additional file 10: Table S2G).* RELA*^*FUS1*^ target genes from 293T-RELA^FUS1^ and mEPN cells were also found in “ST-Radial-Glia-like” and ‘ST-Metabolic’ signature genes, respectively. Taken together, these results suggest that most* RELA*^*FUS1*^ target genes were actively transcribed, thereby driving specific oncogenic pathways necessary for ependymoma formation in significant collaboration with the RELA/NF-κB pathway.

### *RELA*^*FUS1*^ binds on specific DNA regions through the unique DNA-binding motif

**Fig. 3:**
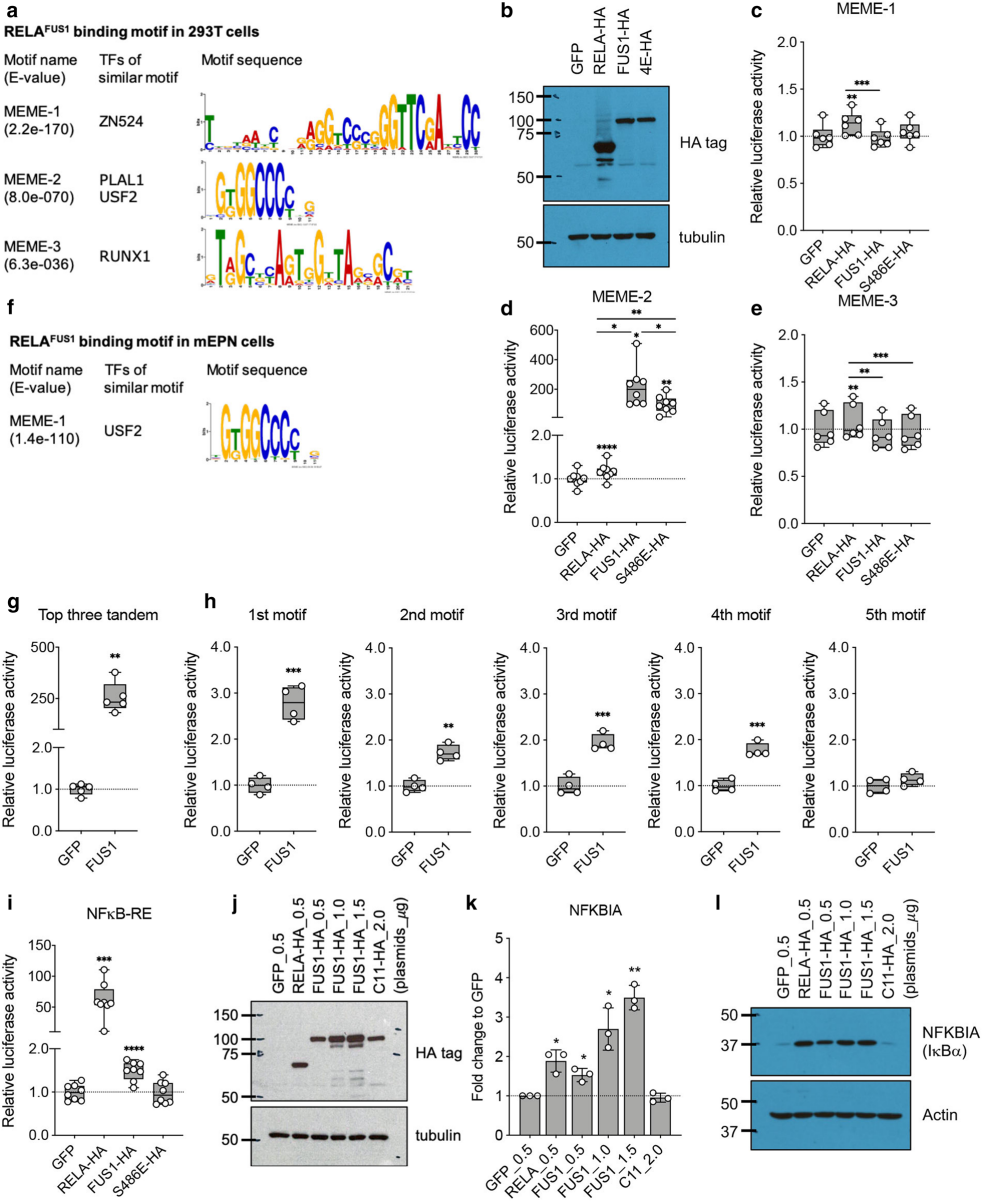
*RELA*^*FUS1*^ binds on specific DNA regions through the unique DNA-binding motif. **a** Top three transcription factor (TF) binding motifs enriched within the* RELA*^*FUS1*^-HA peaks identified by the Multiple Em for Motif Elicitation (MEME) tool in 293T/tv-a cells. E-value, enrichment *p* value. **b** RCAS-GFP, RELA-HA,* RELA*^*FUS1*^-HA (FUS1-HA) and RELA^*FUS1−S486E*^-HA (4E-HA) vector expression in 293T/tv-a cells. **c**–**e** Relative Nanoluc reporter activity to the* RELA*^*FUS1*^-responsive element (RE) normalized to the Firefly luciferase activity and GFP cells in the 293T/tv-a cells (n = 6 or 8, in technical triplicate). MEME-1, 2 and 3 denote* RELA*^*FUS1*^-responsive reporters for top three (3×) or five (5×) tandem* RELA*^*FUS1*^-motif as shown in Additional file 4: Fig. S3D–F. **f** Top TF binding motif enriched within the* RELA*^*FUS1*^-HA peaks in mEPN cells. **g**, **h** Relative Nanoluc reporter activity to the top three tandem (3×) or top five single* RELA*^*FUS1*^-MEME-2 motifs normalized to the Firefly luciferase activity and GFP cells in 293T cells (n = 4 or 5, in technical triplicate). **i** Relative Nanoluc reporter activity to the NF-κB-RE normalized to the Firefly luciferase activity and GFP cells in 293T/tv-a cells (n = 8, in technical triplicate). Analysis was done using RM one-way ANOVA (**c**-**e**) or paired two-tailed t-test (**g**–**i**). *p < 0.05; **p < 0.01; ***p < 0.001; ****p < 0.0001. **j** RCAS vector expression in 293T cells. C11-HA, RCAS-C11orf95-HA **k** Relative NFKBIA mRNA expression in 293T cells. Data (mean ± SD) are displayed as the relative ratio to GFP cells (n = 3, in technical quadruplicate). Analysis was done using paired two-tailed t-test. *p < 0.05; **p < 0.01. **l** IκBα protein expression in 293T cells. See Methods for **j**–**l**

High NF-κB activity mediated by the transcriptional activity of *RELA*^*FUS1*^ is thought to play a critical role in the *RELA*^*FUS1*^-driven ependymoma formation [41, 45]. Although some overlap between* RELA*^*FUS1*^ and Rela target genes were observed, non-Rela target genes were more predominantly identified among the* RELA*^*FUS1*^ target genes (Fig. 2n, o), implying the creation of a unique DNA-binding motif of* RELA*^*FUS1*^, which is independent of RELA/NF-κB regulation. Of note, the RELA/NF-κB dimer can be associated with many non-NF-κB consensus sequences [32, 67]. We thus used the Multiple Em for Motif Elicitation (MEME) Suite to explore what transcription factor (TF) binding motifs are enriched in* RELA*^*FUS1*^ and RELA^*FUS1−S486E*^ ChIP-seq peaks in 293T/tv-a cells (Fig. 1a–d) [1], and identified unique DNA-binding motifs, some of which were shared between them (Fig. 3a and Additional file 4: Fig. S3A). Interestingly, the canonical NF-κB consensus motif, termed as κB site (5′-GGGRNWYYCC-3′, R = A or G, N = any base, W = A or T, and Y = C or T) was not present among the top 10 motifs in either* RELA*^*FUS1*^ or RELA^*FUS1−S486E*^ peaks (Additional file 11: Table S3A) [5, 24, 36]. In turn, when applying the TF motif analysis to Rela Peaks in MEFs [37], the NF-κB-like motif (5′-KGGAAADYCCM-3′, K = G or T, D = A or G or T, M = A or C) was identified in only 17.4% of the Rela target genes as the most enriched motif, thus confirming the presence of Rela binding on non-NF-κB consensus sequence (Additional file 4: Fig. S3B). The* RELA*^*FUS1*^ motifs or any related motifs were not present among the top 5 motifs in Rela peaks (Additional file 11: Table S3B).

To systematically examine whether *RELA*^*FUS1*^ activates the gene expression via DNA-binding motifs identified by ChIP-seq, we generated luciferase reporter constructs for three* RELA*^*FUS1*^ motifs ranked at the top 3 and tested them in 293T/tv-a cells (Fig. 3b–e, Additional file 4: Fig. S3C-F; Additional file 11: Table S3A). We found that* RELA*^*FUS1*^ responded to the* RELA*^*FUS1*^-MEME-2 motif (5′-BGKGGCCCCBG-3′, B = C or G or T) but not to MEME-1 and 3 (Fig. 3c–e; Additional file 11: Table S3C). Further,* RELA*^*FUS1*^ also responded to both the triple tandem of the MEME-2 sequence ranked at 1st to 3rd in the de novo motif discovery analysis and the single MEME-2 sequence ranked within the top 4 when transiently introduced the *RELA*^*FUS1*^ in 293T cells (Fig. 3g, h, Additional file 4: Fig. S3G; Additional file 11: Table S3C). Interestingly, RELA^*FUS1−S486E*^ also evidently reacted to the MEME-2 motif to a somewhat lower degree compared to the* RELA*^*FUS1*^, whereas wild-type RELA barely responded to the MEME-2 motif (Fig. 3d). Of note,* RELA*^*FUS1*^ and RELA^*FUS1−S486E*^ proteins were less expressed than RELA in the 293T/tv-a cells (Fig. 3b), thus emphasizing their actual activities to the MEME-2 motif. Further, when applying the TF motif analysis to mEPN cells (Fig. 2a, b), a very similar GC-rich motif (5′-CNGGGGCCACR-3′) to the 293T-RELA^FUS1^-MEME-2 motif was identified as the top-ranked motif (Fig. 3f; Additional file 11: Table S3D, E). These GC-rich motifs were present in 44.2 (123 out of 278 peaks) and 43.5 (194 out of 446 peaks) percent of all* RELA*^*FUS1*^ peaks in 293T-RELA^FUS1^ and mEPN cells, respectively. Again, no enrichment for the canonical NF-κB consensus motif was detected in the top 10 motifs in mEPN cells (Additional file 11: Table S3D).

To subsequently test if *RELA*^*FUS1*^ is able to directly activate the expression of NF-κB target genes via binding to the NF-κB consensus motif, we used a reporter construct containing the NF-κB responsive element (5 × κB sequence). As expected, expression of wild-type *RELA* strongly activated the reporter system and induced mRNA and protein expression of NFKBIA, a representative NF-κB target gene (Fig. 3i–l), whereas expression of *RELA*^*FUS1*^ only minimally activated the system (Fig. 3i). Nevertheless, forced-expression of *RELA*^*FUS1*^ steadily induced mRNA and protein expression of NFKBIA in a dose-dependent manner, signifying that *RELA*^*FUS1*^ is competent to regulate the NF-κB pathway via the consensus sequence (Fig. 3j–l, Additional file 4: Fig. S3H) [58]. In turn, *RELA*^*FUS1−S486E*^ did not activate the reporter assay (Fig. 3i). These results suggested that* RELA*^*FUS1*^ might form its unique transcription network through the GC-rich MEME-2 motif in collaboration primarily with a yet unknown-NF-κB motif, rather than the consensus NF-κB motif.

### *C11orf95* determines the* RELA*^*FUS1*^-binding region through the unique GC-rich motif

**Fig. 4:**
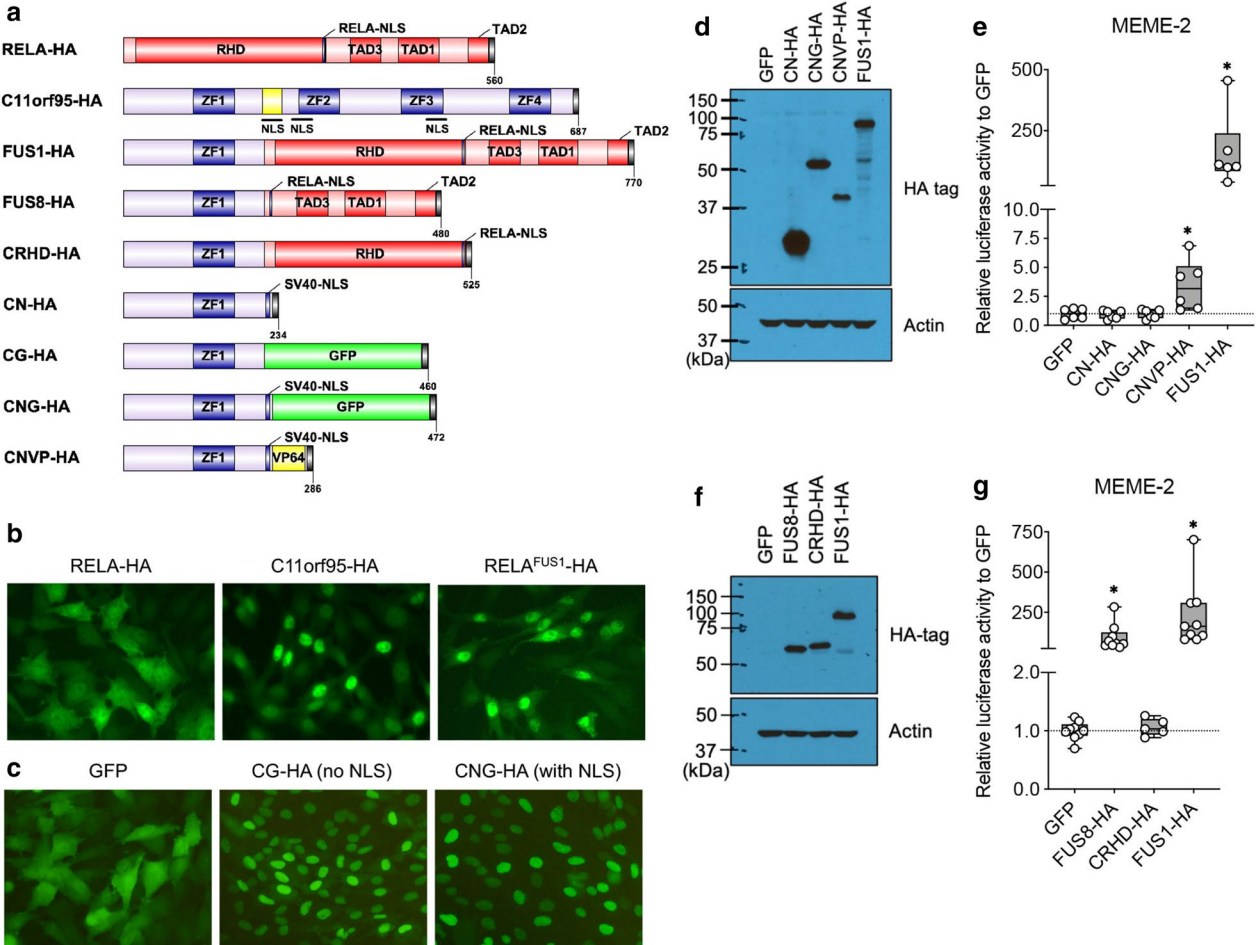
*C11orf95* determines the* RELA*^*FUS1*^-binding region through the unique GC-rich motif. **a** Schematic of RCAS vector constructs (See Methods) **b** Subcellular localization of RELA-HA, C11orf95-HA and* RELA*^*FUS1*^-HA in DF-1 cells. **c** Subcellular localization of GFP and GFP-fusion proteins. DF-1 cells expressing the relevant RCAS virus were observed with fluorescent microscopy. Representative images were shown in **b** and **c**. **d, f** RCAS vectors expression in 293T/tv-a cells. **e, g** Relative Nanoluc reporter activity to the* RELA*^*FUS1*^-RE-luciferase (MEME-2) normalized to the Firefly luciferase activity and GFP cells in the 293T/tv-a cells expressing the indicated RCAS virus as shown in the **d** or **f**. (**e**, n = 6; **g**, n = 5 or 9 in technical triplicate) Analysis was done using paired two-tailed t-test (**e**) or RM one-way ANOVA (**g**). *p < 0.05

The minimal response of RELA to the* RELA*^*FUS1*^-MEME-2 motif leads to the suggestion that the C11orf95 domain rather than RELA might be a critical determinant for the DNA binding of* RELA*^*FUS1*^ proteins to the MEME-2 motif (Fig. 3d).* RELA*^*FUS1*^-HA proteins preferentially localized in the nucleus compared to RELA-HA proteins (Fig. 4a, b) [45]. So far, C11orf95 protein function has not been described well. However, as predicted by multiple C2H2 type zinc finger domains and the putative nuclear localization signal (NLS), the C11orf95 proteins predominantly accumulate in the nucleus (Fig. 4a, b) [22, 45]. Interestingly, the C11orf95 portion (C11ΔC) of the* RELA*^*FUS1*^ was sufficient for the nuclear localization despite the fact that the putative NLS of C11orf95 is lost, implying the presence of an additional NLS (Fig. 4a, c). We thus hypothesized that the C11ΔC portion preserving one zinc finger domain might contribute to nuclear localization and DNA-binding through the unique binding motif of* RELA*^*FUS1*^ proteins, thereby regulating the transcriptional activity of the target genes by the RELA’s activation domain in the* RELA*^*FUS1*^ protein. To reveal the molecular mechanism by which *RELA*^*FUS1*^ regulates the expression of specific target genes with the MEME-2 motif, we generated several *C11orf95* mutants and analyzed their ability to activate the 5xMEME-2 luciferase reporter assay (Fig. 4a). The C11ΔC-NLS (CN-HA) and C11ΔC-NLS-GFP fusion (CNG-HA) were unable to activate the MEME-2 reporter, likely due to the absence of a functional activation domain (Fig. 4d, e). In turn, a construct that replaced the RELA portion of *RELA*^*FUS1*^ with SV40NLS-VP64, in which VP64 is a tetrameric repeat of herpes simplex VP16 minimal activation domain (C11ΔC-NLS-VP64; CNVP-HA), evidently activated the MEME-2 reporter (Fig. 4a, d, e) [2, 47]. In addition, we tested the ability of *RELA*^*FUS8*^ (Type 8)—a naturally occurring variant of *RELA*^*FUS*^, lacking most of the Rel homology domain (RHD)—as well as CRHD-HA, a *RELA*^*FUS1*^ mutant lacking the activation domains in the RELA portion (Fig. 4a, f) [9, 36]. *RELA*^*FUS8*^ strongly activated the MEME-2 reporter at levels comparable to *RELA*^*FUS1*^ (Fig. 4g). In turn, deletion of the RELA activation domain (CRHD-HA) resulted in the inability to activate the MEME-2 reporter (Fig. 4g). Of note, the *RELA*^*FUS8*^ failed to induce brain tumor formation in mice [60], thus signifying that the RHD was indispensable for the tumor-forming potential of *RELA*^*FUS1*^, whereas might be dispensable for the transcriptional activity via the MEME-2 motif. Taken together, these observations suggested that the C11ΔC portion primarily determined the DNA binding loci of* RELA*^*FUS1*^ on the MEME-2 motif. However, the cooperation of both the C11ΔC and RELA portion are essential for the regulation of the transcriptional target genes of *RELA*^*FUS1*^.

### *RELA*^*FUS1*^ transcriptionally regulates the target gene expression through DNA binding on the* RELA*^*FUS1*^-MEME-2 sequence

**Fig. 5:**
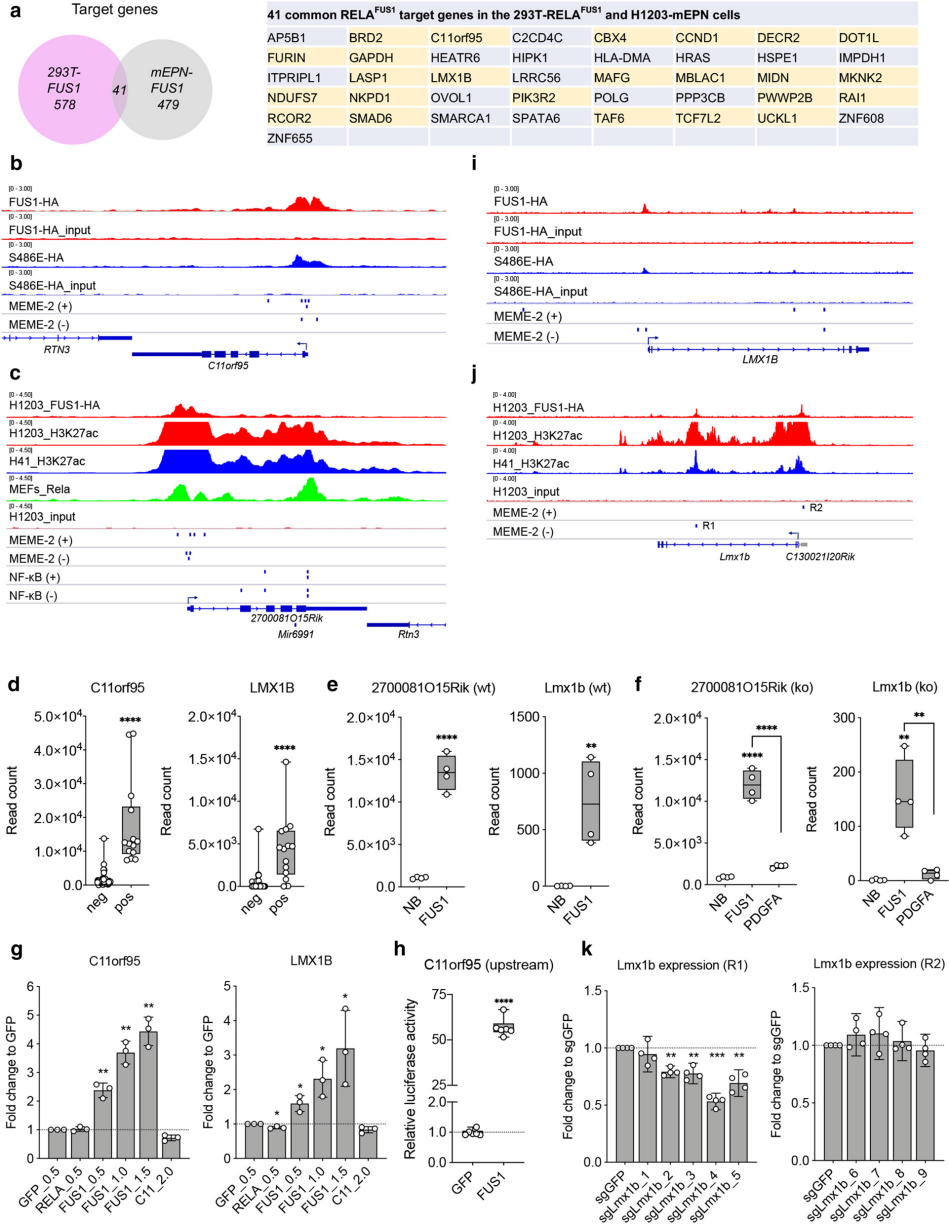
*RELA*^*FUS1*^ transcriptionally regulates the target gene expression through DNA binding on the* RELA*^*FUS1*^-MEME-2 sequence. **a** Venn diagram showing the number of the* RELA*^*FUS1*^ target genes overlapped between 293T and mEPN cells. 41 common targets were shown in the right table. SE-located genes were highlighted in yellow. **b**, **c**, **i**, **j*** RELA*^*FUS1*^-HA,* RELA*^*FUS1−S486E*^-HA and H3K27ac binding profiles surrounding the human *C11orf95* and *LMX1B* in 293T/tv-a, and mouse *2700081O15Rik* and *Lmx1b* loci in mEPN (H1203 and H41) cells. Rela binding profile in MEFs was also shown in mouse *2700081O15Rik* locus. The position of the MEME-2 and κB sites are shown as a blue vertical bar on positive (+) and negative (−) DNA strands. **d** Boxplots of C11orf95 and LMX1B mRNA expression in human *RELA*^*FUS*^ positive (n = 14) and negative (n = 54) ependymomas. **e**, **f** Boxplots of 2700081O15Rik and Lmx1b mRNA expression in mouse normal brain (NB) and RCAS-*RELA*^*FUS1*^-driven ependymoma or *PDGFA*-driven glioma tissues in the *N/tv-a* (**e**) or *N/tv-a;Ink4a-Arf*^*−/−*^*;Pten*^*fl/fl*^ (KO) (**f**) mice (n = 4 in each group). All box plots showing mRNA expression extend from the 25th to 75th percentiles. Whiskers of the box plots extend to the most extreme data point (**d**–**f**). Gene expression analysis was done using unpaired two-tailed t-test (**d**, **e**) or Ordinary one-way ANOVA (**f**). **p < 0.01; ****p < 0.0001. **g** Relative C11orf95 and LMX1B mRNA expression in 293T cells. Data are displayed as the relative ratio to GFP cells. qPCR data (mean ± SD) for C11orf95 and LMX1B expression are displayed as the relative ratio to GFP cells (n = 3, in technical quadruplicate). Analysis was done using paired two-tailed t-test. *p < 0.05; **p < 0.01 (See Methods). **h** Relative Nanoluc reporter activity to the upstream sequence of *C11orf95* gene normalized to the Firefly luciferase activity and GFP cells in the 293T cells (n = 6, in technical triplicate). Analysis was done using paired two-tailed t-test. ****p < 0.0001 **k** Relative Lmx1b mRNA expression in H1203 cells introduced with the indicated dCas-sgRNA. Data are displayed as the relative ratio to sgGFP vector-infected cells. Data from two independent experiments with two technical replicates are shown in the figures. Each qPCR was performed in technical quadruplicate. Analysis was done using paired two-tailed t-test. **p < 0.01; ***p < 0.001

In general, transcription factors regulate the expression of target genes in a context-dependent manner [24]. In fact, L1 cell adhesion molecule (L1CAM), a well-known downstream marker of *RELA*^*FUS*^ was identified as a* RELA*^*FUS1*^ target gene in 293T-*RELA*^*FUS1*^ but not in mEPN cells (Additional file 5: Fig. S4A–D) [45]. Therefore, we examined the overlap between* RELA*^*FUS1*^ ChIP-seq peaks in 293T-*RELA*^*FUS1*^ and mEPN cells (Fig. 1a, 2a). Unexpectedly, we found that only 41 genes were shared between these cells, implying a flexible DNA-binding capacity of *RELA*^*FUS*^ as a TF function, depending on the cellular context (Fig. 5a; Additional file 9: Table S1E). However, it is noteworthy that *CCND1*, *H-Ras*, *PIK3R2*, and *DOT1L* in addition to *C11orf95* were identified in the common target genes. 24 out of the 41 shared genes were located within SE regions, presumably serving as core target genes responsible for *RELA*^*FUS1*^-driven ependymoma formation (Fig. 2k, 5a and Additional file 5: Fig. S4E–G).

The fact that *C11orf95* was a* RELA*^*FUS1*^ target in both cell types provides significant insights into the understanding of the oncogenic mechanism of *RELA*^*FUS1*^. Prominent peaks of* RELA*^*FUS1*^ binding were detected around the TSS within both the *C11orf95* and *2700081O15Rik* loci in the HA ChIP-seq analyses (Fig. 5b, c). Interestingly, multiple MEME-2 sites were concomitantly found in the* RELA*^*FUS1*^ peaks, thus strongly suggesting a direct transcriptional regulation of *C11orf95* gene expression by *RELA*^*FUS1*^ (Fig. 5b, c). Indeed, human *RELA*^*FUS*^ positive ependymomas exhibited significantly higher C11orf95 mRNA expression than negative ones (Fig. 1e, 5d and Additional file 2: Fig. S1F). Further, as bolstered by the overlapping of H3K27ac peaks with the* RELA*^*FUS1*^ peaks in the *2700081O15Rik* gene (Fig. 5c), *2700081O15Rik* was remarkably up-regulated in *RELA*^*FUS1*^-driven ependymomas relative to normal brains and *PDGFA*-driven gliomas (Fig. 5e, f). Forced-expression of *RELA*^*FUS1*^ in 293T cells was able to induce C11orf95 mRNA expression in a dose-dependent manner (Fig. 3j, 5g). Further, to confirm if *RELA*^*FUS1*^ had a direct impact on the *C11orf95* expression, we cloned an immediate upstream sequence of the *C11orf95* gene, including three MEME-2 motifs into a luciferase reporter vector (Additional file 4: Fig. S3C, Additional file 6: Fig. S5A). We found that the expression of *RELA*^*FUS1*^ strongly activated this reporter, suggesting that *RELA*^*FUS1*^ is able to up-regulate its own expression directly (Fig. 5h). Since *RELA*^*FUS*^ is caused by the genomic rearrangement involving *C11orf95* and *RELA* loci [45], the expression of the *RELA*^*FUS*^ gene is thought to be controlled by the *C11orf95* promoter, thus possibly resulting in the formation of an autoregulatory feedback loop in the tumors (Additional file 6: Fig. S5B). Of note, *2700081O15Rik* was found to be a direct Rela target gene, as shown by a Rela peak with multiple κB sites in the gene locus, which are different from MEME-2 sites (Fig. 5c; Additional file 9: Table S1E). However, the lack of* RELA*^*FUS1*^ binding to the κB sites suggests a specific *2700081O15Rik* gene regulation by *RELA*^*FUS*^ independent of Rela.

The distribution of* RELA*^*FUS1*^ binding loci in our ChIP-seq analyses suggests that *RELA*^*FUS1*^ might also control the gene expression via an intronic enhancer regulatory region. Thus, to further investigate the TF function of *RELA*^*FUS1*^, we focused on the *LMX1B* gene, a brain-developmental transcription factor, which was one of the common* RELA*^*FUS1*^ target genes in 293T-*RELA*^*FUS1*^ and mouse ependymoma cells and was also selected as one of the enhancer-associated genes specific for human* RELA*^*FUS*^ tumors and mouse ependymoma cells (Fig. 2k, 5a) [31].* RELA*^*FUS1*^ bound similar positions on the *LMX1B* gene locus in 293T and mouse ependymoma cells. Two* RELA*^*FUS1*^ peaks containing the MEME-2 motif were found in the putative promoter region (upstream of the TSS) and second long intron of both human and mouse genes (Fig. 5i, j). The H3K27ac peaks were also concomitantly identified in these loci in mEPN cells, suggesting the direct gene regulation by *RELA*^*FUS1*^ (Fig. 5j). As expected, a significant upregulation of *LMX1B* was observed in both human and mouse* RELA*^*FUS*^ tumors (Fig. 1e, 5d–f). Further, forced-expression of *RELA*^*FUS1*^ in 293T cells was able to induce *LMX1B* expression in a dose-dependent manner (Fig. 3j, 5g). Interestingly, GeneHancer profiling indicated an association between the promoter and enhancer regulatory elements in the second intron of the human *LMX1B* gene (Additional file 6: Fig. S5C) [8]. Therefore, to dissect a mechanism of *LMX1B* gene regulation by* RELA*^*FUS1*^, we examined if physical perturbation of* RELA*^*FUS1*^ binding on these loci affected the gene expression using the CRISPR-dCas9 system [49]. We designed multiple sgRNAs to target the CRISPR-dCAS9-sgRNA complexes in two regions around the MEME-2 motif denoted as Region-1 and -2 (R1 and R2), and then lentivirally introduced them in mEPN cells (Fig. 5j, Additional file 6: Fig. S5D-F). Targeting R1 (intronic region) but not R2 (promoter region) resulted in significant downregulation of *Lmx1b* gene expression (Fig. 5k, Additional file 6: Fig. S5G). Collectively, these findings appear to represent prototypic examples for epigenetic gene regulation of *RELA*^*FUS1*^, thus confirming the oncogenic TF function of *RELA*^*FUS1*^ on the MEME-2 sequence.

### Anti-cancer drug screening highlights oncogenic signaling driven by* RELA*^*FUS1*^ target genes

**Fig. 6:**
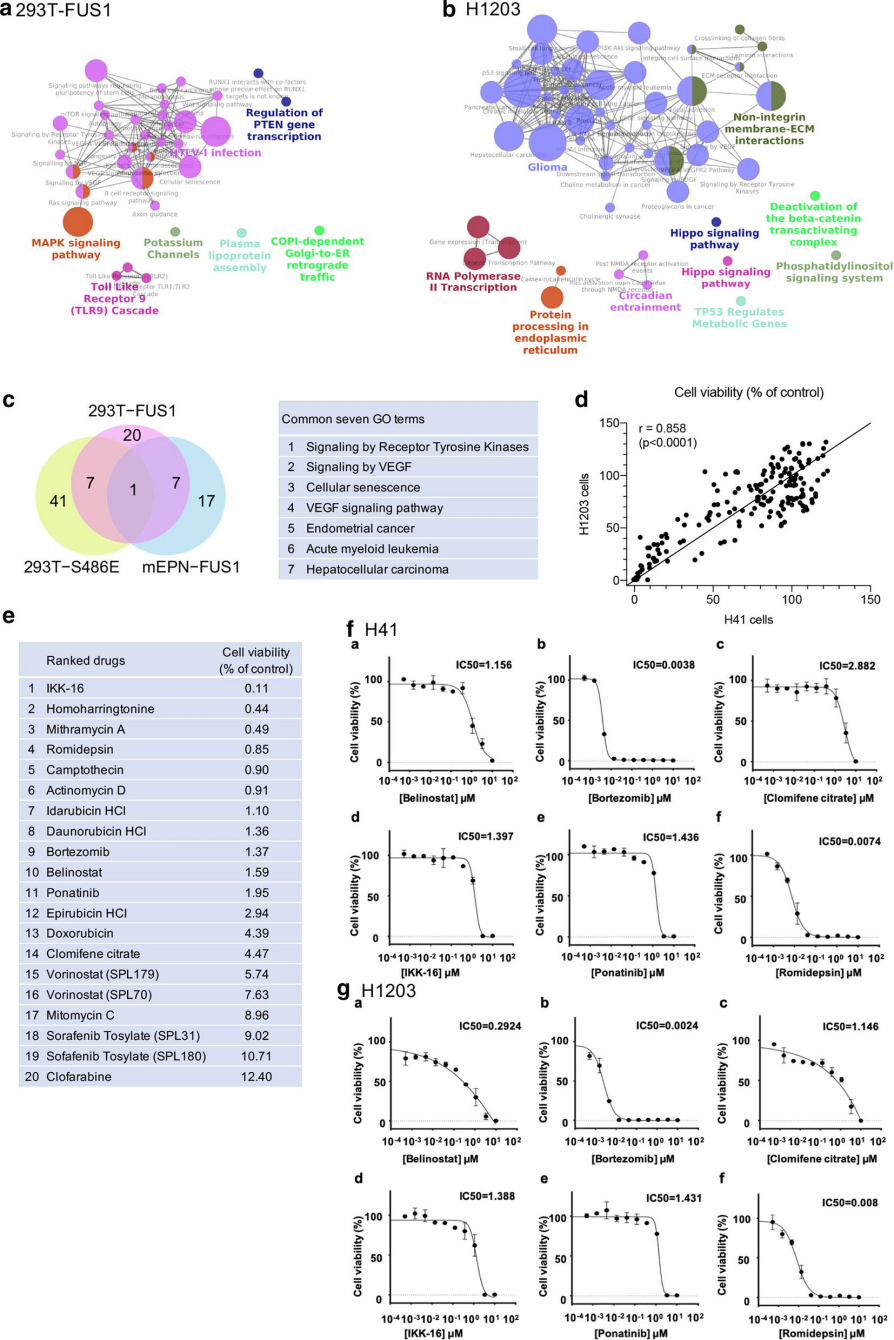
Anti-cancer drug screening highlights oncogenic signaling driven by* RELA*^*FUS1*^ target genes. **a**, **b** Pathway enrichment analysis for* RELA*^*FUS1*^ target genes in 293T-*RELA*^*FUS1*^ (**a**) and H1203-mEPN (**b**) cells. Color nodes and the size represent the enriched gene set and the number of genes in each gene set, respectively. **c** Venn diagram showing the number of the overlapping of pathways involved with the 293T-*RELA*^*FUS1*^, 293T-*RELA*^*FUS1−S486E*^, and H1203-RELA^FUS1^ target genes. Common GO term of dysregulated pathways driven by* RELA*^*FUS1*^ target genes within the TSS ± 10 kb in 293T-*RELA*^*FUS1*^ and H1203 cells is shown in the right panel. **d** Correlation of cell viability in two mEPN cells (H41 and H1203). Cells were treated with 179 anti-cancer drugs with 10 μM for 72 h in two technical replicates. The mean values of two independent experiments for each cells are shown in x and y axis, respectively. Analysis was done using two-tailed Pearson's correlation. **e** List of twenty drugs presented over 85% growth inhibition relative to the 0.1% DMSO-treated control. The mean values of cell viability in H41 and H1203 cells are shown in the figure. **f**, **g** Cell viability of mEPN cells (**f** H41 and **g** H1203 cells) treated with six selected drugs. The IC50 values of each anti-cancer drug were shown in the figures

We finally used Gene Ontology (GO) analysis based on our ChIP-seq experiments to explore the signaling network directly regulated by* RELA*^*FUS1*^ target genes. As expected by the small number of* RELA*^*FUS1*^ target genes shared between 293T-*RELA*^*FUS1*^ and mEPN cells, diverse signaling networks were enriched in these cells with a little overlap between both cell types (Figs. 5a, 6a, b) [3]. Target genes of the 293T-*RELA*^*FUS1*^ were notably involved in the MAPK signaling pathway, signaling pathways regulating pluripotency of stem cells, VEGF signaling, and Regulation of PTEN gene transcription (Fig. 6a; Additional file 12: Table S4A). On the other hand, GO terms enriched in mEPN cells converged to Glioma, PI3K-Akt signaling pathway, Signaling by PDGF, VEGF signaling pathway, RNA Polymerase II Transcription, Protein processing in the endoplasmic reticulum, and non-integrin membrane-ECM interactions (Fig. 6b; Additional file 12: Table S4B). Target genes of 293T-*RELA*^*FUS1−S483E*^ presented somewhat different signaling pathways from those of 293T-*RELA*^*FUS1*^ as suggested by the number of target genes shared between these cells, presumably due to an impairment of DNA and/or protein binding due to the mutation in the RHD (Figs. 1g, h, 6a, Additional file 7: Fig. S6A, Additional file 12: Table S4A, C). Interestingly, when focusing on common pathways between the 293T-*RELA*^*FUS1*^ and mEPN cells, we identified an enrichment of ‘Signaling by Receptor Tyrosine Kinases (RTKs)’, particularly VEGF signaling (Fig. 6c). Association of aberrant RTK activity, such as EFN, PDGF, and FGFR signaling, with ependymomagenesis was previously reported [19, 31, 41, 44] and thereby implicated as potential therapeutic targets for this tumor type (Additional file 7: Fig. S6B, C, Additional file 12: Table S4D-G). Further, PDGF signaling was found to be a direct transcriptional target of *RELA*^*FUS1*^ in mEPN cells (Additional file 7: Fig. S6D), consistent with previous observations that PDGF signaling was up-regulated in human and mouse* RELA*^*FUS1*^ tumors [41, 44, 45]. Therefore, to examine if blockade of these signaling pathways had a significant inhibitory effect on the tumor growth, we performed an anti-cancer drug screening using the FDA-approved drug library with additional selected-NF-κB inhibitors in two mEPN cells (Additional file 3: Fig. S2A; Additional file 13: Table S5A) [40]. We treated the cells with these drugs and focused on drugs presented over 85% growth inhibition (Fig. 6d, e, Additional file 8: Fig. S6E; Additional file 13: Table S5B). As expected, multi-tyrosine kinase inhibitors such as Sorafenib (targeting VEGFR, PDGFR and RAF) and Ponatinib (targeting BCR-ABL, Src, VEGFR, FGFR, and PDGFR) were able to effectively inhibit the growth of these cells (Fig. 6e; Additional file 13: Table S5B) [38, 66]. Interestingly, in addition to an IκB kinase inhibitor (IKK-16), HDAC inhibitors (Belinostat, Romidepsin, Vorinostat) and a Proteasome inhibitor (Bortezomib), both of which were known to block NF-κB signaling were able to effectively inhibit the growth of mEPN cells, likely supporting the contribution of NF-κB activity in* RELA*^*FUS1*^ ependymomas [26, 33, 62]. We then determined the half-maximal inhibitory concentration values (IC50) of these drugs and selected six representatives from these drug categories. The IC50 values of these drugs were very similar between both mEPN cells, indicating the high specificity of these inhibitors to* RELA*^*FUS1*^ (Fig. 6f, g). These results suggest that inhibitors against NF-κB and RTK signaling (most notably drugs targeting VEGFR and PDGFR) could be promising therapeutic agents for* RELA*^*FUS*^ tumors.

## Discussion

In this study, we performed ChIP-seq experiments to explore target genes that are directly regulated by *RELA*^*FUS1*^ and unveiled the unique epigenetic regulation of *RELA*^*FUS*^ in ependymoma formation (Additional file 8: Fig. S6F). Activation of the NF-κB pathway has been so far well-documented in human and mouse* RELA*^*FUS*^ ependymomas [41, 45]. However, it remained to be determined how this pathway contributes to tumorigenesis and if it might serve as an actual therapeutic target in patients. Our findings suggest that *RELA*^*FUS1*^ might induce brain tumor formation through two main oncogenic pathways regulated by C11orf95 and RELA target genes (Additional file 8: Fig. S6F). The NF-κB pathway driven by the RELA portion was essential for tumorigenesis, although unknown-RELA/NF-κB target pathways were likely more common than canonical pathways via the κB site. Thus, blockade of active NF-κB pathways will likely be one option for *RELA*^*FUS*^ positive ependymoma therapy.

*RELA* regulates NF-κB target genes by forming homo- or heterodimers, and the selectivity of the NF-κB response is variable according to the dimerization partner [23, 57]. Of note, the RELA/NF-κB dimer can interact with both NF-κB consensus motif and many non-consensus sequences [32, 67]. It is not known whether* RELA*^*FUS*^ forms dimers or if the dimerization is necessary for tumorigenesis. However, our reporter assays with the MEME-2 and NF-κB motifs and the absence of the κB site in the DNA sequences bound by* RELA*^*FUS1*^ indicate that DNA binding of* RELA*^*FUS1*^ might have deviated from that of RELA, thus suggesting unusual dimerization of* RELA*^*FUS1*^.

The *RELA*^*FUS1−S486E*^ mutant remarkably responded to the MEME-2 motif but completely failed to recognize the κB site. Phosphorylation of S276 in the RHD by PKAc induces the conformation change of RELA and subsequent recruitment of p300/CBP, thereby resulting in promoting the transcriptional activity [71]. Thus, severe impairment of the oncogenicity of the mutant might be explained by the inability to exert a precise conformation change, consequently losing the capacity to activate the NF-κB pathway. Similarly, the *RELA*^*FUS8*^ variant, which is lacking the RHD, was capable of recognizing the MEME-2 motif and displayed prominent transcriptional activity but failed to induce brain tumors [60]. These observations support that both RELA/NF-κB activity and binding to the MEME-2 motif on its own is insufficient but essential for the tumor-forming potential of *RELA*^*FUS1*^.

We observed* RELA*^*FUS1*^ binding peaks concomitant with MEME-2 sequences, which is recognized by the C11orf95 portion of *RELA*^*FUS1*^, within the regulatory regions of* RELA*^*FUS1*^ target genes. The RELA subunit preferentially binds to a DNA sequence consisting of a series of G bases at the 5′ position followed by a central A/T base for fine transcriptional regulation [10, 36, 63]. Therefore, the absence of the central A/T bases, characteristic for the consensus NF-κB motif sequence, in the MEME-2 motif strongly suggests that transcriptional regulation of these C11orf95 target genes by *RELA*^*FUS1*^ was independent of gene regulation via the consensus NF-κB motif [63]. Interestingly, we have recently shown that *RELA*^*FUS*^ variants with one C2H2 type zinc finger domain in the C11orf95 portion (*RELA*^*FUS1*^ and *RELA*^*FUS4*^) presented a more aggressive phenotype compared to those with two zinc finger domains (*RELA*^*FUS2*^ and *RELA*^*FUS3*^), supporting the importance of the *RELA*^*FUS*^ function via the C2H2 type zinc finger domain [60]. Taken together, these observations suggested that two independent programs driven by C11orf95 and RELA target genes are likely central players in the tumorigenic functions of *RELA*^*FUS*^.

Our anti-cancer drug screening highlighted several compounds targeting signaling pathways associated with the oncogenic mechanisms of *RELA*^*FUS1*^, such as RTK, HDAC and NF-κB inhibitors including a proteasome inhibitor. Interestingly, Actinomycin D, previously identified as a potential drug for* RELA*^*FUS*^ tumors, was also selected in the top-ranked drugs, thus supporting a specificity of our screening to the* RELA*^*FUS*^ [61]. Further, a Phase II Clinical Trial of Marizomib, a second-generation irreversible proteasome inhibitor is currently ongoing for “Recurrent Low-Grade and Anaplastic Supratentorial, Infratentorial and Spinal Cord Ependymoma” (NCT03727841). The therapeutic effect is predicted especially against* RELA*^*FUS*^ tumors. However, it is of note that such a screening is generally biased toward identifying cytotoxic agents, as also demonstrated by our screening. Thus, a more careful selection would be essential for precisely evaluating the specificity of compounds.

Our understanding of the molecular mechanisms underlying the *RELA*^*FUS*^-driven ependymoma formation is increasingly deepened by recent key reports [31, 72]. In this study, we not only successfully reproduced many of the data shown in the previous studies but also presented a more detailed functional analysis of *RELA*^*FUS*^ in several aspects [31, 72]. Our analyses similarly identified *L1CAM*, *IGF2*, *C11orf95*, *DOT1L, CCND1* and *RELA* genes as direct* RELA*^*FUS*^ targets that contain the specific* RELA*^*FUS*^ binding motif. Further, we experimentally demonstrated the transcriptional regulation of target genes by *RELA*^*FUS*^ and highlighted a potential autoregulatory feedback loop by *RELA*^*FUS*^ of itself in these tumors. Interestingly, several* RELA*^*FUS*^ target genes such as *L1CAM* and *IGF2* were not necessarily common in 293T and mouse ependymoma cells in our analyses, indicating the fact that the transcriptional programs activated by *RELA*^*FUS*^ are context-dependent. Thus, our study emphasizes the significance to examine TF functions in various experimental settings. In contrast to gene fusions involving protein kinases, such as the *BCR-ABL* fusion in chronic myeloid leukemia [21], therapeutic targets for cancers harboring gene fusion involving transcription factors are more elusive owed to their complex functions, as shown in this study. Therefore, our experimental approach integrating with ChIP-seq, RNA-seq, functional studies, and drug screenings will be helpful for not only a better understanding of ependymoma biology but also the identification of the precise therapeutic targets. Ependymomas are still lethal brain tumors, and hopefully, the results of this study will greatly contribute to the advancement of ependymoma research.

## Conclusions

So far, the contribution of the C11orf95 moiety to *RELA*^*FUS*^-driven ependymoma formation has been considerably underestimated. Our study revealed that the C11orf95 moiety was a key determinator for the nuclear localization and DNA binding of* RELA*^*FUS*^, consequently forming the complex oncogenic signaling networks in significant collaboration with the RELA targets. These findings will provide therapeutic insights for patients with *RELA*^*FUS*^ positive ependymoma.

## Supplementary Information

Additional file1.

Additional file2 (JPG 497 KB)

Additional file3 (JPG 458 KB)

Additional file4 (JPG 472 KB)

Additional file5 (JPG 442 KB)

Additional file6 (JPG 752 KB)

Additional file7 (JPG 620 KB)

Additional file8 (JPG 217 KB)

Additional file9.

Additional file10.

Additional file11.

Additional file12.

Additional file13.

Additional file14.

## Data Availability

ChIP-seq data have been deposited in the DNA Data Bank of Japan (DDBJ) with the accession numbers DRA010686. All codes in this study are available upon request.

## Abbreviations

*RELA*^*FUS*^: *C11orf95-RELA* fusion
*RELA*^*FUS1*^: *C11orf95-RELA* type 1 fusion
mEPN: mouse ependymoma
SE: super-enhancer
ST-EPN: supratentorial ependymoma
TF: transcription factor
TSS: transcription start site
