# Publisher Correction to: *C11orf95-RELA* fusion drives aberrant gene expression through the unique epigenetic regulation for ependymoma formation

**DOI:** 10.1186/s40478-021-01157-y

**Published:** 2021-05-27

**Authors:** Tatsuya Ozawa, Syuzo Kaneko, Frank Szulzewsky, Zhiwei Qiao, Mutsumi Takadera, Yoshitaka Narita, Tadashi Kondo, Eric C. Holland, Ryuji Hamamoto, Koichi Ichimura

**Affiliations:** 1 Division of Brain Tumor Translational Research, National Cancer Center Research Institute, Chuo-ku, Tokyo, 104-0045 Japan; 2 Division of Molecular Modification and Cancer Biology, National Cancer Center Research Institute, Chuo-ku, Tokyo, 104-0045 Japan; 3 Human Biology Division, Fred Hutchinson Cancer Research Center, 1100 Fairview Avenue North, Mailstop C3-168, Seattle, WA 98109 USA; 4 Division of Rare Cancer Research, National Cancer Center Research Institute, Chuo-ku, Tokyo, 104-0045 Japan; 5 Department of Neurosurgery, Yokohama City University, Yokohama, Kanagawa 236-0027 Japan; 6 Department of Neurosurgery and Neuro-Oncology, National Cancer Center Hospital, Chuo-ku, Tokyo, 104-0045 Japan; 7 Seattle Tumor Translational Research Center, Fred Hutchinson Cancer Research Center, 1100 Fairview Avenue North, Seattle, WA 98109 USA; 8 RIKEN Center for Advanced Intelligence Project, Cancer Translational Research Team, 1-4-1 Nihonbashi, Chuo-ku, Tokyo, 103-0027 Japan

## Correction to: Ozawa et al. acta neuropathol commun (2021) 9:36 https://doi.org/10.1186/s40478-021-01135-4

In the original publication of the article [1] there were errors introduced during the publication process involving the italic emphasis. These errors are as followed:

- The italicized terms of “*RELA*^*FUS*^” have been amended as this was erroneously applied to the genes and proteins.
