# Supplementary material for: C11orf95-RELA fusion drives aberrant gene expression through the unique epigenetic regulation for ependymoma formation
Source: Acta Neuropathol Commun. 2021 Mar 8;9:36. doi: 10.1186/s40478-021-01135-4 (PMC7941712; doi:10.1186/s40478-021-01135-4)
Supplement: Supplementary file 1 — Additional file1. [file 40478_2021_1135_MOESM1_ESM.docx]

**Additional file 1:** **SUPPLEMENTAL INFORMATION**

***C11orf95-RELA* fusion drives aberrant gene expression through the unique epigenetic regulation for ependymoma formation**

Tatsuya Ozawa, Syuzo Kaneko, Frank Szulzewsky, Zhiwei Qiao, Mutsumi Takadera, Yoshitaka Narita, Tadashi Kondo, Eric C. Holland, Ryuji Hamamoto, Koichi Ichimura

**Table of Contents:**

Supplementary Figures S1-S6

Supplementary Figure legends

Supplementary Table legends

**Additional file 2:** **Figure S1** **related to Figure 1. HA-tag ChIP-seq analyses identified unique genomic binding sites of RELA^FUS1^.** (A) Experimental workflow for identifying RELA^FUS1^ target genes (B) Tv-a-myc protein expression in 293T/tv-a cells. Cell lysates of 293T cells lentivirally infecting the tv-a-myc or GFP were subjected to immunoblot analysis with the indicated antibodies. (C) RCAS-GFP, C11orf95-RELA-HA type1 (FUS1-HA) and C11orf95-RELA-HA type1 (S486E-HA) vector expression in 293T/tv-a cells. Cell lysates of cells retrovirally infecting the relevant RCAS viruses were subjected to immunoblot analysis with the indicated antibodies. (D) Schematic of RELA and RELA^FUS1-S486E^ protein (See Methods). Phosphorylation of Ser-276 within the Rel homology of RELA has been reported to promote RELA transcriptional activity [2, 8]. Nevertheless, the phospho-mimetic mutant with serine-to-glutamine substitution at Ser-486 of RELA^FUS1^ corresponding to Ser-276 in the Rel homology domain (RHD) of RELA has severely impaired the transforming capacity of *RELA^FUS1^* [11]. (E) Heatmap for the HA ChIP-seq dataset presented a considerable correlation between the biological replicate. (F) Volcano plot illustrating differences in gene expression between ST-EPN-RELA and YAP1 subgroups (FDR < 0.05, n = 11,786) [13]. Differences in Log2 fold change in gene expression values are plotted on the x-axis. Adjusted p-values calculated using the Benjamin-Hochberg method are plotted on the y-axis. RELA^FUS1^ target genes annotated within the TSS±10kb in 293T-RELA^FUS1^ cells are shown as large circles. *C11orf95, RELA, CCND1* and *L1CAM* genes are indicated by the arrows.

**Additional file 3:** **Figure S2 related to Figure 2. Most RELA^FUS1^ target genes are actively transcribed.** (A) RELA^FUS1^ and RELA^FUS1^-HA protein expression in mouse ependymoma cell lines, which were established from the brain tumors induced with the RCAS-RELA^FUS1^ (H41, 57 and 59) or RELA^FUS1^-HA (H1203) in *N/tv-a; Ink4a-arf^-/-^; Pten^fl/fl^* mice. NS1 and NS2 denote neurosphere lines derived from normal pups forebrain of *N/tva; Ink4a-arf^-/-^; Pten^fl/fl^* or *B/tv-a* mouse, respectively. Cell lysates of these cells were subjected to immunoblot analysis with the indicated antibodies to confirm the RELA^FUS1^ or RELA^FUS1^-HA protein expression. White and black arrows show RELA^FUS1^ and endogenous RELA proteins, respectively. (B) H3K27ac ChIP-seq analyses in H41-mEPN cells. The enrichment of H3K27ac within the RELA^FUS1^-HA binding region (see Fig. 2A) is shown in heatmaps. (C and D) Venn diagrams for the number of the overlapping H3K27ac peaks (C) and super-enhancers (D) in mEPN (H41 and H1203) cells are shown separately, using two replicates. (E and F) Venn diagram showing the number of the overlapping of enhancer (E) and SE (F) -annotated genes between mouse ependymoma cells (H41 and H1203) and human RELA^FUS^ tumors [10]. (G-I) Venn diagram showing the number of the overlapping between the previously reported-NF-κB target genes [5, 11] and Rela target genes in MEFs (G), RELA^FUS1^ target genes in 293T- RELA^FUS1^ (H) or RELA^FUS1^ target genes in mEPN cells (I).

**Additional file 4:** **Figure S3 related to Figure 3. RELA^FUS1^ binds on specific DNA regions through the unique DNA-binding motif.** (A) Top three transcription factor binding motifs enriched within the RELA^FUS1-S486E^-HA peaks identified by the Multiple Em for Motif Elicitation (MEME) tool in 293T/tv-a cells. *E*-value, enrichment *p*-value. (B) Rela binding motif most enriched within the Rela peaks identified by the MEME tool in TNF-treated MEFs. *E*-value, enrichment *p*-value. (C-F) Schematic of a luciferase reporter construct to measure a Nanoluc reporter activity through the RELA^FUS1^ motif (C). Top three or five RELA^FUS1^ binding motif sequences for MEME-1 (D), 2 (E) and 3 (F) motifs were inserted in the upstream of a minimal promoter (blue box) in tandem, respectively. The DNA sequences for each motif used in the reporter assay are shown below the sequence logos, respectively. (G) RCAS vector expression in 293T cells. To measure Nanoluc reporter activity through the RELA^FUS1^-MEME-2 motif sequence, RCAS-GFP or RELA^FUS1^-HA vector with the relevant luciferase reporter vectors was transiently transfected in 293T cells. After 24 hours of the transfection, the cell lysates were subjected to luciferase reporter assay and immunoblot analysis with the indicated antibodies. (H) RELA^FUS1^-HA and RELA^FUS1-S486E^-HA binding profiles surrounding the human *NFKBIA* locus in 293T/tv-a cells. The position of the κB site was shown as a blue vertical bar on positive (+) and negative (-) DNA strands. MEME-2 site was not identified in this locus.

**Additional file 5:** **Figure S4 related to Figure 5. *L1CAM* and *CCND1* are direct transcriptional target genes of *RELA^FUS1^*.**

(A) Boxplots of L1CAM and CCND1 mRNA expression in human *RELA^FUS^* positive (n = 14) and negative (n = 54) ependymomas. (B, E and F) RELA^FUS1^-HA, RELA^FUS1-S486E^-HA, H3K27ac and Rela binding profiles surrounding the human *L1CAM* (B) and *CCND1* (E) gene locus in the 293T/tv-a cells, and mouse *Ccnd1* (F) gene locus in mEPN cells and MEFs. RELA^FUS1^-HA, RELA^FUS1-S486E^-HA, H3K27ac, Rela and input peaks are shown with the same scale in each figure. The position of the 293T-RELA^FUS1^-MEME-2 and κB site is shown as a blue vertical bar on positive (+) and negative (-) DNA strands. Representative images in two technical replicates were shown in the figure. (C) Relative L1CAM and CCND1 mRNA expression in 293T cells. qPCR data (mean ± SD) for L1CAM and CCND1 expression are displayed as the relative ratio to GFP cells (n = 3, in technical quadruplicate). Analysis was done using paired two-tailed t-test. * = p < 0.05, ** = p < 0.01, *** = p < 0.001. (D) L1CAM protein expression in 293T cells. RCAS vectors were transiently transfected with the indicated plasmid concentration to adjust to the similar protein expression level between samples. After 48hrous of the transfection, cell lysates were subjected to immunoblot analysis with the indicated antibodies. (G) Boxplots of mouse Ccnd1 mRNA expression in mouse normal brain (NB) and RCAS-*RELA^FUS1^*-driven ependymoma or *PDGFA*-driven glioma tissues in the indicated genetic background (n = 4 in each group). All box plots showing mRNA expression extend from the 25th to 75th percentiles (A and G). Whiskers of the box plots extend to the most extreme data point. Gene expression analysis was done using unpaired two-tailed t-test (A and G left panel) or Ordinary one-way ANOVA (G right panel). ** = p < 0.01, *** = p < 0.001, **** = p < 0.0001.

L1 cell adhesion molecule (L1CAM) and CCND1 (cyclin D1) were identified as downstream targets up-regulated in *RELA^FUS^* positive ependymomas (Fig. 1e, S1F and S4A) and their specific immunopositivity with that of RELA is commonly used as a potential surrogate marker for RELA^FUS^ ependymomas in the clinic [4, 12, 14, 17]. However, the molecular mechanism underlying the increased expression of these proteins in RELA^FUS1^ tumors remains to be fully determined. *L1CAM* was not selected as a common RELA^FUS1^ target gene between the 293T-RELA^FUS1^ and mEPN cells in the HA ChIP-seq analyses, indicating a cell or species-type-specific epigenetic regulation by *RELA^FUS1^* (Fig. 5a). However, two significant RELA^FUS1^ peaks containing the RELA^FUS1^-MEME-2 sequence were detected around the TSS in 293T-RELA^FUS1^ and RELA^FUS1-S486E^ cells (Fig. S4B). We thus examined if *RELA^FUS1^* could drive *L1CAM* gene expression and found that forced expression of *RELA^FUS1^* in 293T cells drastically increased endogenous L1CAM transcription, an increase that was also observed at the protein level in a dose-dependent manner (Fig. S4C and D), thus evidently indicating that *L1CAM* is a direct transcriptional target gene of *RELA^FUS1^*.

*CCND1*, a well-known NF-κB target gene, was selected as a common RELA^FUS1^ target gene in our ChIP-seq analyses (Fig. 5a, S4E, F and Table S1E) [6, 7]. Significant peaks of RELA^FUS1^ were detected with the MEME-2 sequence in the upstream and/or downstream of the TSS in both 293T-RELA^FUS1^ and mEPN cells (Fig. S4E and F). High-level CCND1 protein expression was observed in human and mouse RELA^FUS^ tumors (Fig. S4A and G). As expected, forced-expression of *RELA^FUS1^* into 293T cells significantly induced CCND1 mRNA expression in a dose-dependent manner, thus supporting a direct transcriptional regulation by RELA^FUS1^ (Fig. S4C). The protein encoded by this gene plays a critical role in cell cycle control commonly in both normal and cancer cells [6, 7, 18]. Thus, *CCND1* is thought to be a direct transcriptional target gene of *RELA^FUS1^*. However, high expression of the *CCND1* might not be necessarily reliable as a specific surrogate marker to identify *RELA^FUS1^* positive ependymomas as shown by the higher mRNA expression in *PDGFA*-driven gliomas (Fig. S4G, right panel).

**Additional file 6:** **Figure S5 related to Figure 5. *RELA^FUS1^* transcriptionally regulates the target gene expression through DNA binding on the RELA^FUS1^-MEME-2 sequence.** (A) Upstream proximal sequence of the *C11orf95* gene. Purple open arrows indicate RELA^FUS1^-MEME-2 sequences serving as potential RELA^FUS1^ binding sites. Black arrow as well as brown triangle show a putative transcription start site (TSS) of *C11orf95* gene [16]. The upstream proximal sequence as highlighted in pink bar was inserted in the pNL3.2 Nanoluc luciferase reporter vector as shown in Fig. S3C. Schematic of the upstream sequence of the *C11orf95* gene was generated using Benchling [Biology Software] [1]. (B) Schematic representation of the putative mechanism for *C11orf95* gene regulation by *RELA^FUS^* (C) Schematic representation of human *LMX1B* gene locus assembled on UCSC Genome Browser (GRCh37/hg19). The GeneHancer profiling indicated an association between the promoter and enhancer regulatory element in the second intron of human *LMX1B* gene as shown in the orange dotted line [3, 9, 15]. Vertical red lines indicate the genomic position of the 293T-RELA^FUS1^-MEME-2 sequences in the locus. (D) Schematic representation of mouse *Lmx1b* gene locus assembled on UCSC Genome Browser (NCBI37/mm9) [9]. The sgRNA target sites and 293T-RELA^FUS1^-MEME-2 motifs are shown as vertical blue and red lines, respectively. (E) Relative Lmx1b mRNA expression in mouse neurosphere established from *N/tva; Ink4a-arf^-/-^; Pten^fl/fl^* and *B/tv-a* mouse pups brain, and mouse ependymoma-derived cells (H1203, H41, H57 and H59). See also Fig. S2A. Data (mean ± SD) are displayed as the relative ratio to *N/tva; Ink4a-arf^-/-^; Pten^fl/fl^* neurosphere sample (n = 1, in technical quadruplicate). (F) dCas9 protein expression in mEPN cells (H1203). dCas9-sgRNA complexes were lentivirally introduced in the mEPN cells. After puromycin selection, cell lysates of these cells were subjected to immunoblot analysis with the indicated antibodies. (G) Schematic representation of the putative mechanism for *Lmx1b* gene regulation by *RELA^FUS^*.

**Additional file 7 and 8:** **Figure S6 related to Figure 6. Anti-cancer drug screening highlights oncogenic signaling driven by RELA^FUS1^ target genes.** (A) Pathway enrichment analysis for RELA^FUS1-S486E^ target genes in 293T/tv-a cells. Color nodes and the size represent the enriched gene set and the number of genes in each gene set, respectively. (B and C) Pathway enrichment analysis for ST-EPN-RELA (B) and YAP1 (C) subgroups. DEGs between the human ST-EPN-RELA and YAP1 tumors were subjected to the pathway enrichment analysis for each subgroup. Color nodes and the size represent the enriched gene set and the number of genes in each gene set, respectively. Significant enrichment of PDGF and RTK signalings in human ST-EPN-RELA tumors was also reproduced in our analysis as demonstrated by the previous study [13]. (D) RELA^FUS1^, Rela and H3K27ac binding profiles surrounding the mouse *Pdgfa, Pdgfb, Pdgfra*, and *Pdgfrb* gene loci in mEPN cells or MEFs. All peaks are shown with the same scale in each panel. *Pdgfa*, *Pdgfb* and *Pdgfrb* were found to be direct transcriptional target genes of *RELA^FUS1^* in mEPN cells. Whilst, *Pdgfb*, *Pdgfra* and *Pdgfrb* were Rela target genes (Table S1E). The position of the 293T-RELA^FUS1^-MEME-2 and κB site is shown as a blue vertical bar on positive (+) and negative (-) DNA strands. Representative images in two technical replicates were shown in the figure. (E) Correlation of cell viability (% of control) between first and second screening in mEPN (H41 and H1203) cells. Cells were treated with 179 anti-cancer drugs with 10 μM for 72 hours, and their cell viability was then evaluated. Means of two independent experiments in each technical duplicate are shown in x (1st screening) and y (2nd screening) axis. Analysis was done using two-tailed Pearson's correlation. (F) Schematic of the putative oncogenic mechanism of *RELA^FUS^*-driven ependymoma formation.

**SUPPLEMENTARY TABLE LEGENDS**

**Additional file 9:** **Table S1.** (A) Summary of ChIP-seq analysis in 293T/tv-a and mEPN cells. (B) List of RELA^FUS1^-HA peaks in TSS±10kb in 293T/tv-a cells (779 peaks; 619 unique genes). (C) List of RELA^FUS1-S486E^-HA peaks in TSS±10kb in 293T/tv-a cells (566 peaks; 446 unique genes). (D) List of RELA^FUS1^ or RELA^FUS1-S486E^ specific target genes bound in TSS ±10kb. Red and blue letters denote RELA^FUS1^ and RELA^FUS1-S486E^ target genes, respectively. (E) List of RELA^FUS1^, RELA^FUS1-S486E^ and Rela target genes bound in TSS±10kb.

**Additional file 10:** **Table S2.** (A) List of RELA^FUS1^-HA peaks in TSS±10kb in H1203-mEPN cells (649 peaks; 520 genes). (B) List of H3K27ac peaks in H41-mEPN cells. (C) List of H3K27ac peaks in H1203-mEPN cells. (D and E) List of super-enhancers (SEs) identified in H3K27ac peaks in mEPN cells (D, H41 and E, H1203 cells). (F) List of overlapping of super-enhancers (SEs) in H41 and H1203-mEPN cells. (G) List of overlapping genes between the single-cell transcriptomic signature genes of ST-ependymomas and the RELA^FUS1^ target genes in 293T-RELA^FUS1^ or mouse ependymoma (H1203) cells

**Additional file 11:** **Table S3.** (A) List of top 10 RELA^FUS1^ motifs in RELA^FUS1^ peaks in 293T-RELA^FUS1^ cells. (B) List of top 5 Rela motifs in Rela peaks in MEFs (C) List of DNA sequences identified as the MEME-2 sequence in 293T-RELA^FUS1^ target genes. (D) List of top 10 RELA^FUS1^ motifs in RELA^FUS1^ peaks in H1203-EPN cells. (E) List of DNA sequences identified as the most enriched motif in H1203-RELA^FUS1^ target genes.

**Additional file 12:** **Table S4.** (A) Dysregulated pathways driven by RELA^FUS1^ target genes in TSS±10kb in 293T-RELA^FUS1^ cells. (B) Dysregulated pathways driven by RELA^FUS1^ target genes in TSS ±10kb in H1203-mEPN cells. (C) Dysregulated pathways driven by RELA^FUS1-S486E^ target genes in TSS ±10kb in 293T-RELA^FUS1-S486E^ cells. (D) Up-regulated genes in ST-EPN-RELA relative to ST-EPN-YAP1 subgroup (DEGs: RELAup vs YAP1, logFC>1, FDR < 0.05) (E) Down-regulated genes in ST-EPN-RELA relative to ST-EPN-YAP1 subgroup (DEGs: RELA vs YAP1up, logFC < -1, FDR < 0.05) (F) Up-regulated pathways in ST-EPN-RELA subgroup (G) Up-regulated pathways in ST-EPN-YAP1 subgroup

**Additional file 13:** **Table S5.** (A) List of 179 drugs used in anti-cancer drug screening. (B) Result of the drug screening in H41- and H1203-mEPN cells. The mean values of cell viability (% of control) in two mEPN cells are shown in the table.

**Additional file 14:** **Table S6.** (A-C) List of vector constructs (A) and primers (B) and antibodies (C).

**SUPPLEMENTARY REFERENCES**

1 Benchling (2020) [Biology Software]. Retrieved from <https://benchlingcom>:

2 Chen LF, Greene WC (2004) Shaping the nuclear action of NF-kappaB. Nature reviews Molecular cell biology 5: 392-401 Doi 10.1038/nrm1368

3 Fishilevich S, Nudel R, Rappaport N, Hadar R, Plaschkes I, Iny Stein T, Rosen N, Kohn A, Twik M, Safran Met al (2017) GeneHancer: genome-wide integration of enhancers and target genes in GeneCards. Database (Oxford) 2017: Doi 10.1093/database/bax028

4 Gessi M, Giagnacovo M, Modena P, Elefante G, Gianno F, Buttarelli FR, Arcella A, Donofrio V, Diomedi Camassei F, Nozza Pet al (2019) Role of Immunohistochemistry in the Identification of Supratentorial C11ORF95-RELA Fused Ependymoma in Routine Neuropathology. The American journal of surgical pathology 43: 56-63 Doi 10.1097/PAS.0000000000000979

5 Gilmore TD Retrieved from <http://www.bu.edu/nf-kb/gene-resources/target-genes/>

6 Guttridge DC, Albanese C, Reuther JY, Pestell RG, Baldwin AS, Jr. (1999) NF-kappaB controls cell growth and differentiation through transcriptional regulation of cyclin D1. Molecular and cellular biology 19: 5785-5799 Doi 10.1128/mcb.19.8.5785

7 Hinz M, Krappmann D, Eichten A, Heder A, Scheidereit C, Strauss M (1999) NF-kappaB function in growth control: regulation of cyclin D1 expression and G0/G1-to-S-phase transition. Molecular and cellular biology 19: 2690-2698 Doi 10.1128/mcb.19.4.2690

8 Huang B, Yang XD, Lamb A, Chen LF (2010) Posttranslational modifications of NF-kappaB: another layer of regulation for NF-kappaB signaling pathway. Cellular signalling 22: 1282-1290 Doi 10.1016/j.cellsig.2010.03.017

9 Kent WJ, Sugnet CW, Furey TS, Roskin KM, Pringle TH, Zahler AM, Haussler D (2002) The human genome browser at UCSC. Genome Res 12: 996-1006 Doi 10.1101/gr.229102

10 Mack SC, Pajtler KW, Chavez L, Okonechnikov K, Bertrand KC, Wang X, Erkek S, Federation A, Song A, Lee Cet al (2018) Therapeutic targeting of ependymoma as informed by oncogenic enhancer profiling. Nature 553: 101-105 Doi 10.1038/nature25169

11 Ozawa T, Arora S, Szulzewsky F, Juric-Sekhar G, Miyajima Y, Bolouri H, Yasui Y, Barber J, Kupp R, Dalton Jet al (2018) A De Novo Mouse Model of C11orf95-RELA Fusion-Driven Ependymoma Identifies Driver Functions in Addition to NF-kappaB. Cell reports 23: 3787-3797 Doi 10.1016/j.celrep.2018.04.099

12 Pages M, Pajtler KW, Puget S, Castel D, Boddaert N, Tauziede-Espariat A, Picot S, Debily MA, Kool M, Capper Det al (2019) Diagnostics of pediatric supratentorial RELA ependymomas: integration of information from histopathology, genetics, DNA methylation and imaging. Brain Pathol 29: 325-335 Doi 10.1111/bpa.12664

13 Pajtler KW, Witt H, Sill M, Jones DT, Hovestadt V, Kratochwil F, Wani K, Tatevossian R, Punchihewa C, Johann Pet al (2015) Molecular Classification of Ependymal Tumors across All CNS Compartments, Histopathological Grades, and Age Groups. Cancer cell 27: 728-743 Doi 10.1016/j.ccell.2015.04.002

14 Parker M, Mohankumar KM, Punchihewa C, Weinlich R, Dalton JD, Li Y, Lee R, Tatevossian RG, Phoenix TN, Thiruvenkatam Ret al (2014) C11orf95-RELA fusions drive oncogenic NF-kappaB signalling in ependymoma. Nature 506: 451-455 Doi 10.1038/nature13109

15 Stelzer G, Rosen N, Plaschkes I, Zimmerman S, Twik M, Fishilevich S, Stein TI, Nudel R, Lieder I, Mazor Yet al (2016) The GeneCards Suite: From Gene Data Mining to Disease Genome Sequence Analyses. Curr Protoc Bioinformatics 54: 1 30 31-31 30 33 Doi 10.1002/cpbi.5

16 Suzuki A, Kawano S, Mitsuyama T, Suyama M, Kanai Y, Shirahige K, Sasaki H, Tokunaga K, Tsuchihara K, Sugano Set al (2018) DBTSS/DBKERO for integrated analysis of transcriptional regulation. Nucleic acids research 46: D229-D238 Doi 10.1093/nar/gkx1001

17 Torre M, Alexandrescu S, Dubuc AM, Ligon AH, Hornick JL, Meredith DM (2019) Characterization of molecular signatures of supratentorial ependymomas. Mod Pathol: Doi 10.1038/s41379-019-0329-2

18 Zhang Q, Sakamoto K, Wagner KU (2014) D-type Cyclins are important downstream effectors of cytokine signaling that regulate the proliferation of normal and neoplastic mammary epithelial cells. Mol Cell Endocrinol 382: 583-592 Doi 10.1016/j.mce.2013.03.016
