# Supplementary figures and images for: C11orf95-RELA fusion drives aberrant gene expression through the unique epigenetic regulation for ependymoma formation
Source: Acta Neuropathol Commun. 2021 Mar 8;9:36. doi: 10.1186/s40478-021-01135-4 (PMC7941712; doi:10.1186/s40478-021-01135-4)

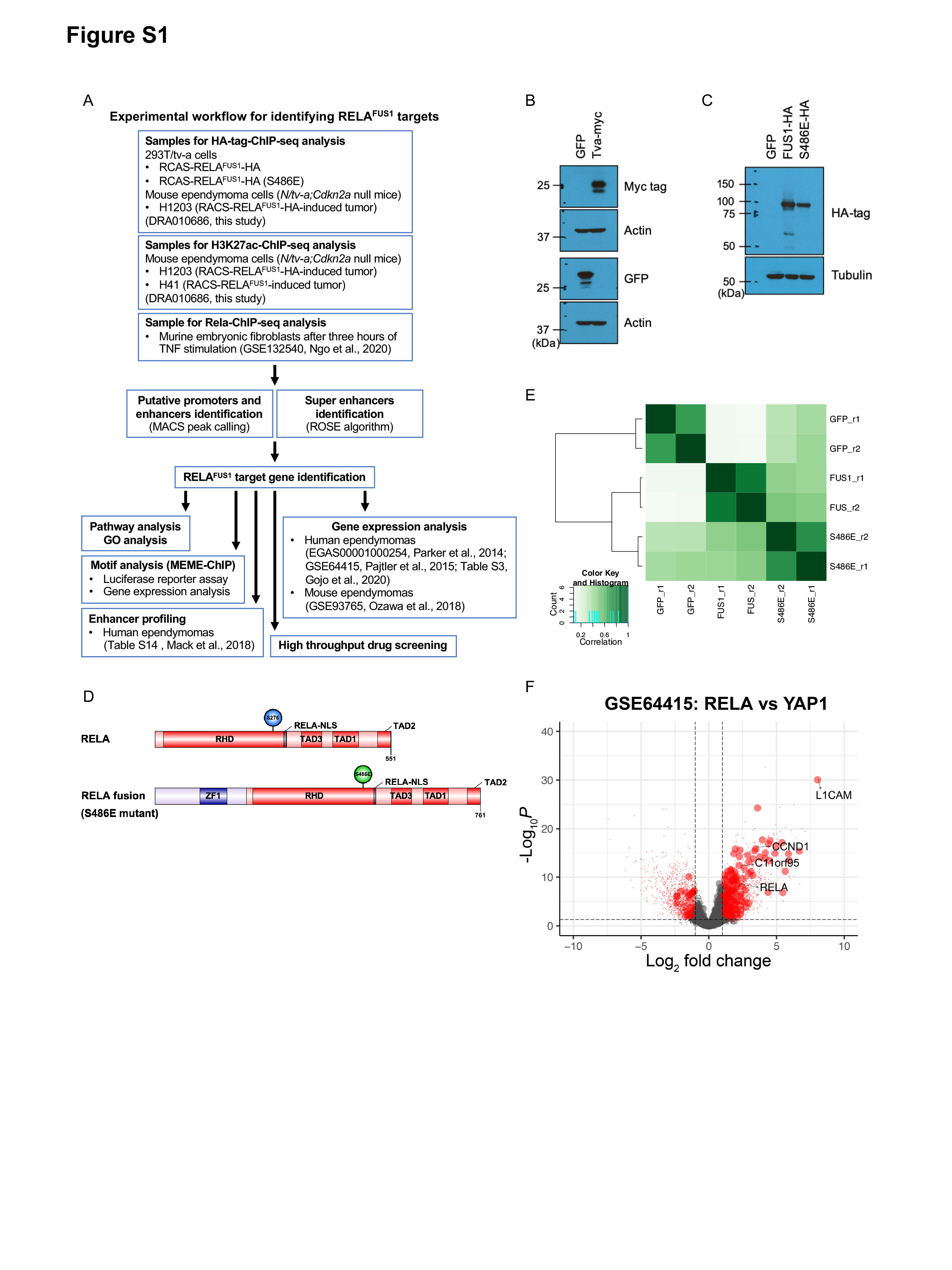

Supplement: Supplementary file 2 — Additional file2 (JPG 497 KB) [file 40478_2021_1135_MOESM2_ESM.jpg]

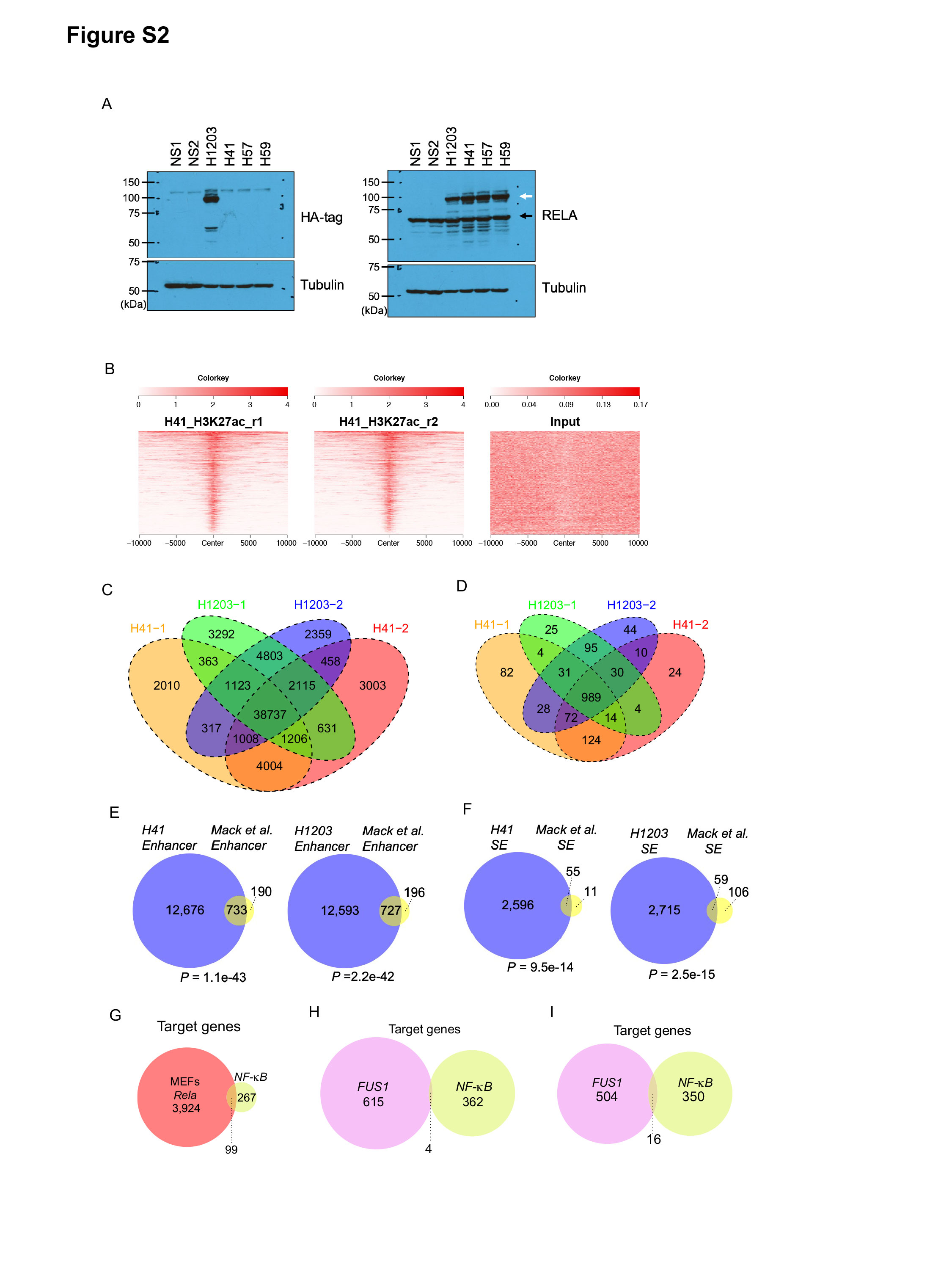

Supplement: Supplementary file 3 — Additional file3 (JPG 458 KB) [file 40478_2021_1135_MOESM3_ESM.jpg]

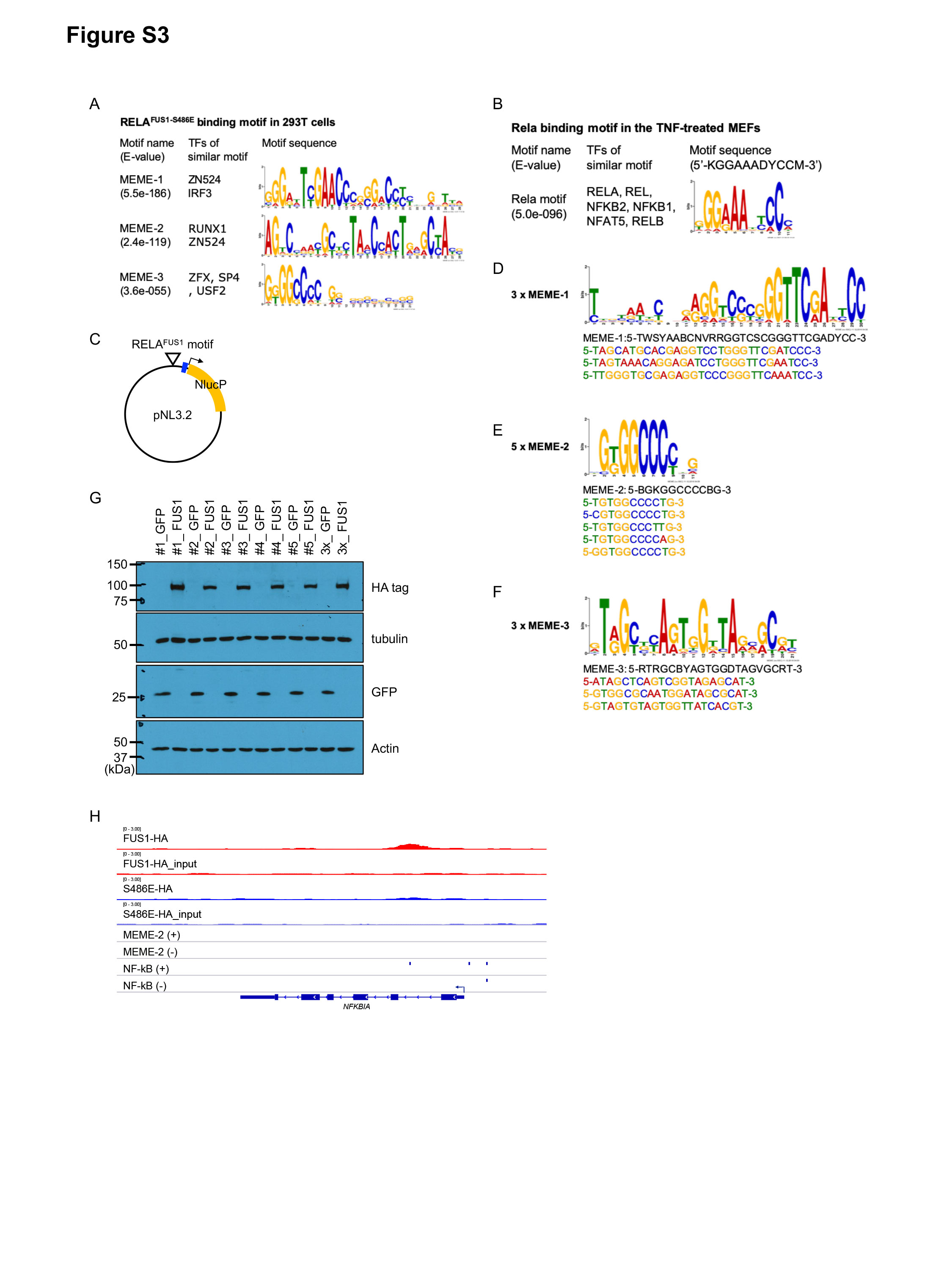

Supplement: Supplementary file 4 — Additional file4 (JPG 472 KB) [file 40478_2021_1135_MOESM4_ESM.jpg]

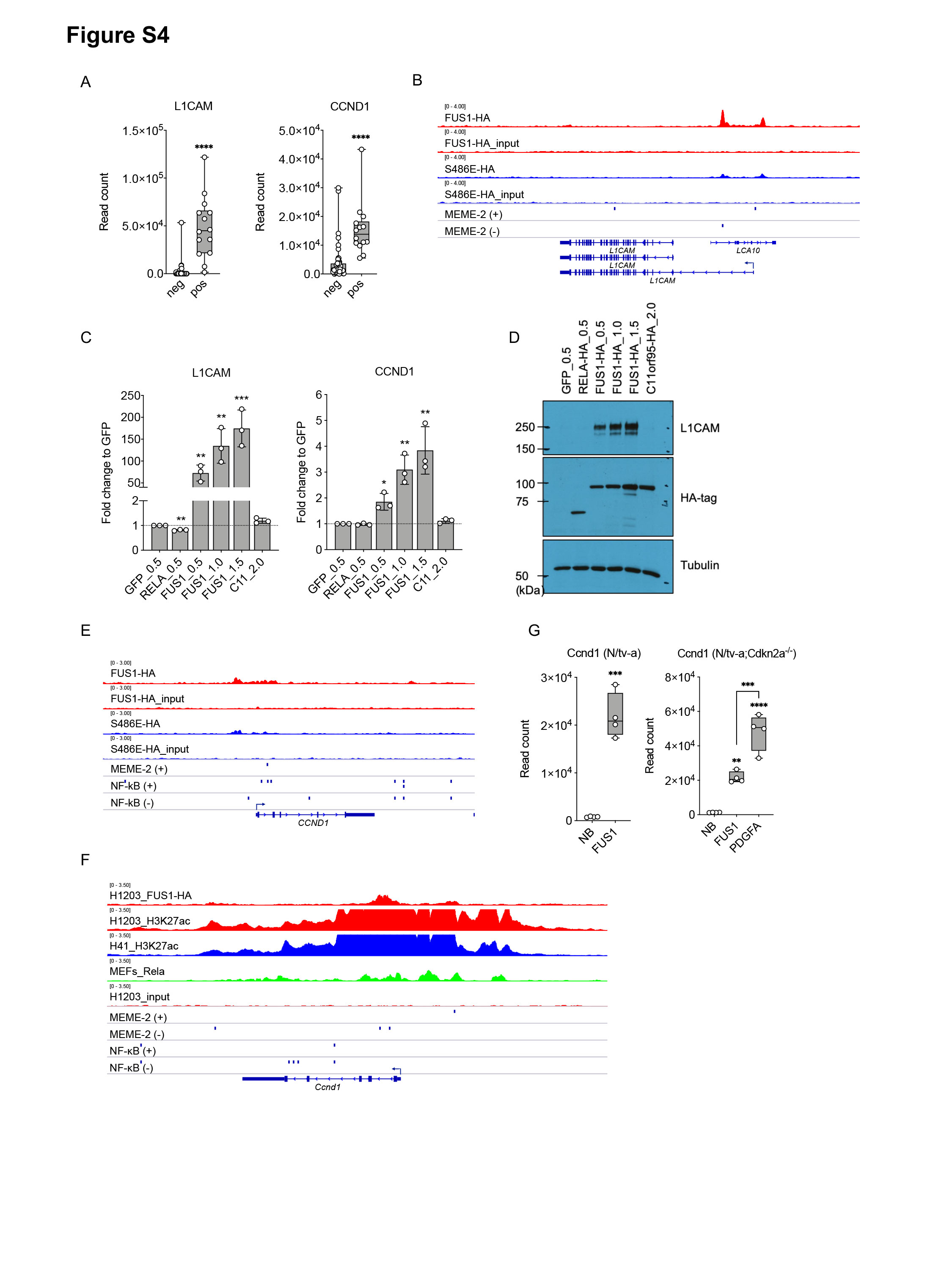

Supplement: Supplementary file 5 — Additional file5 (JPG 442 KB) [file 40478_2021_1135_MOESM5_ESM.jpg]

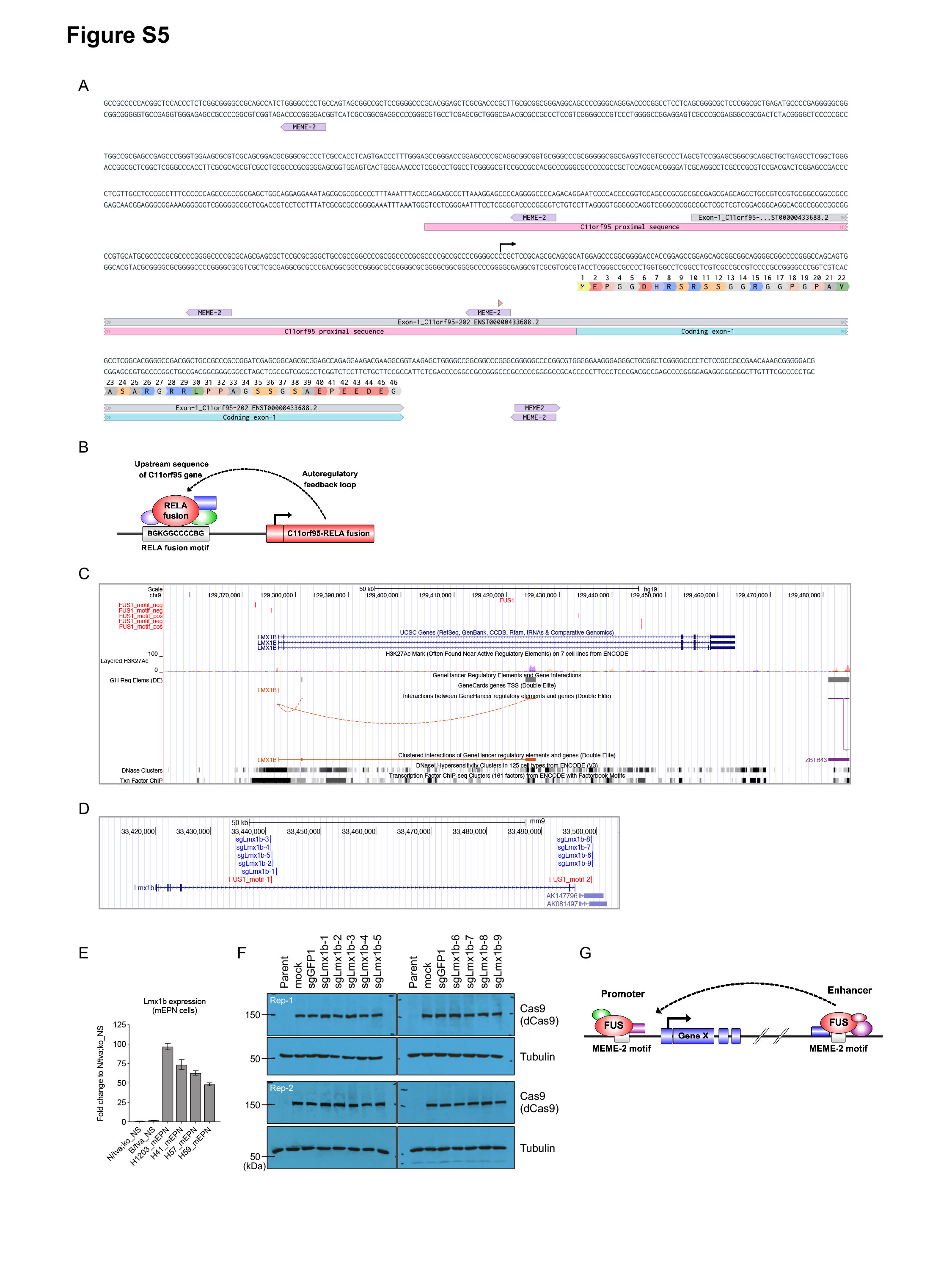

Supplement: Supplementary file 6 — Additional file6 (JPG 752 KB) [file 40478_2021_1135_MOESM6_ESM.jpg]

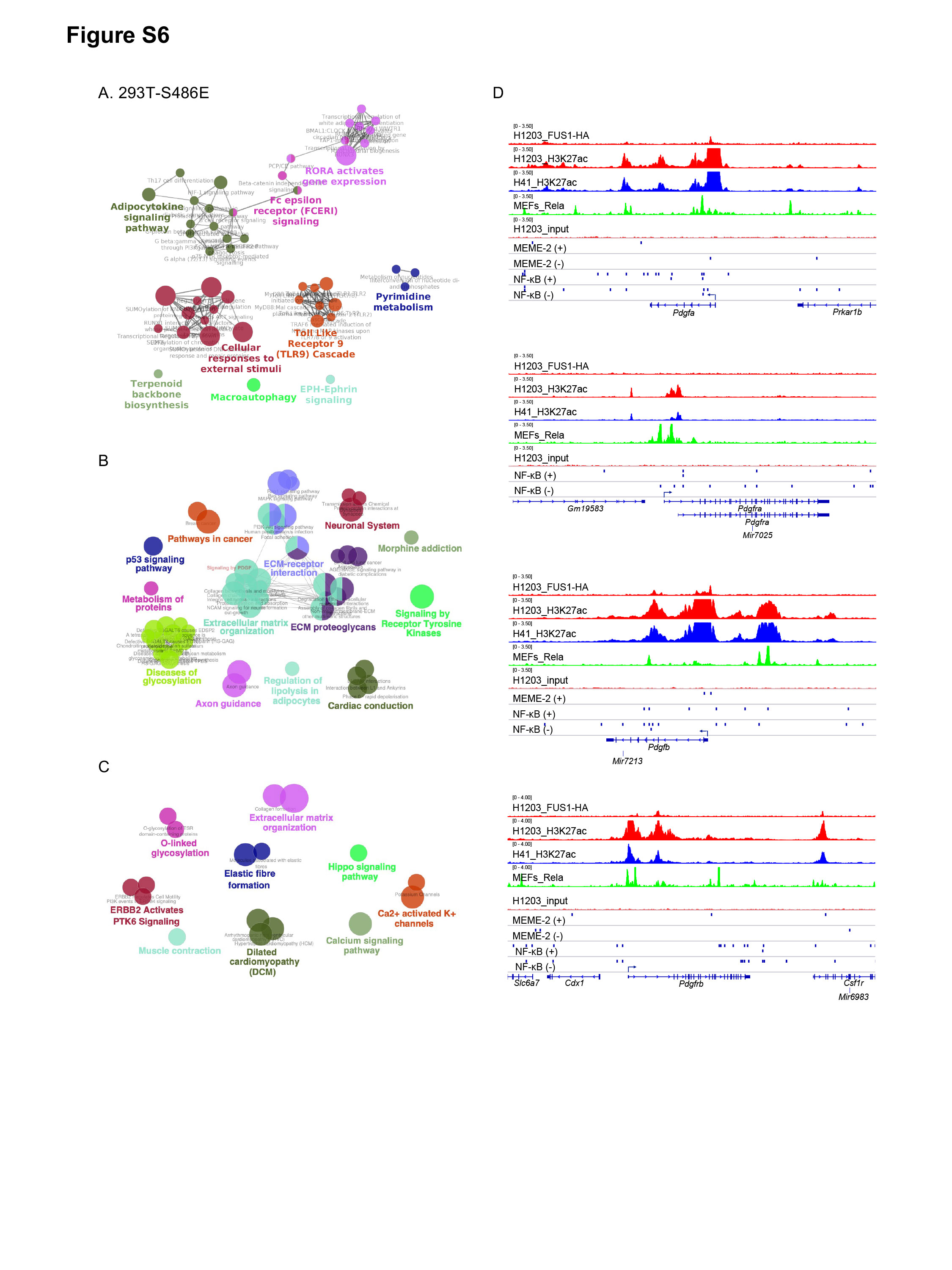

Supplement: Supplementary file 7 — Additional file7 (JPG 620 KB) [file 40478_2021_1135_MOESM7_ESM.jpg]

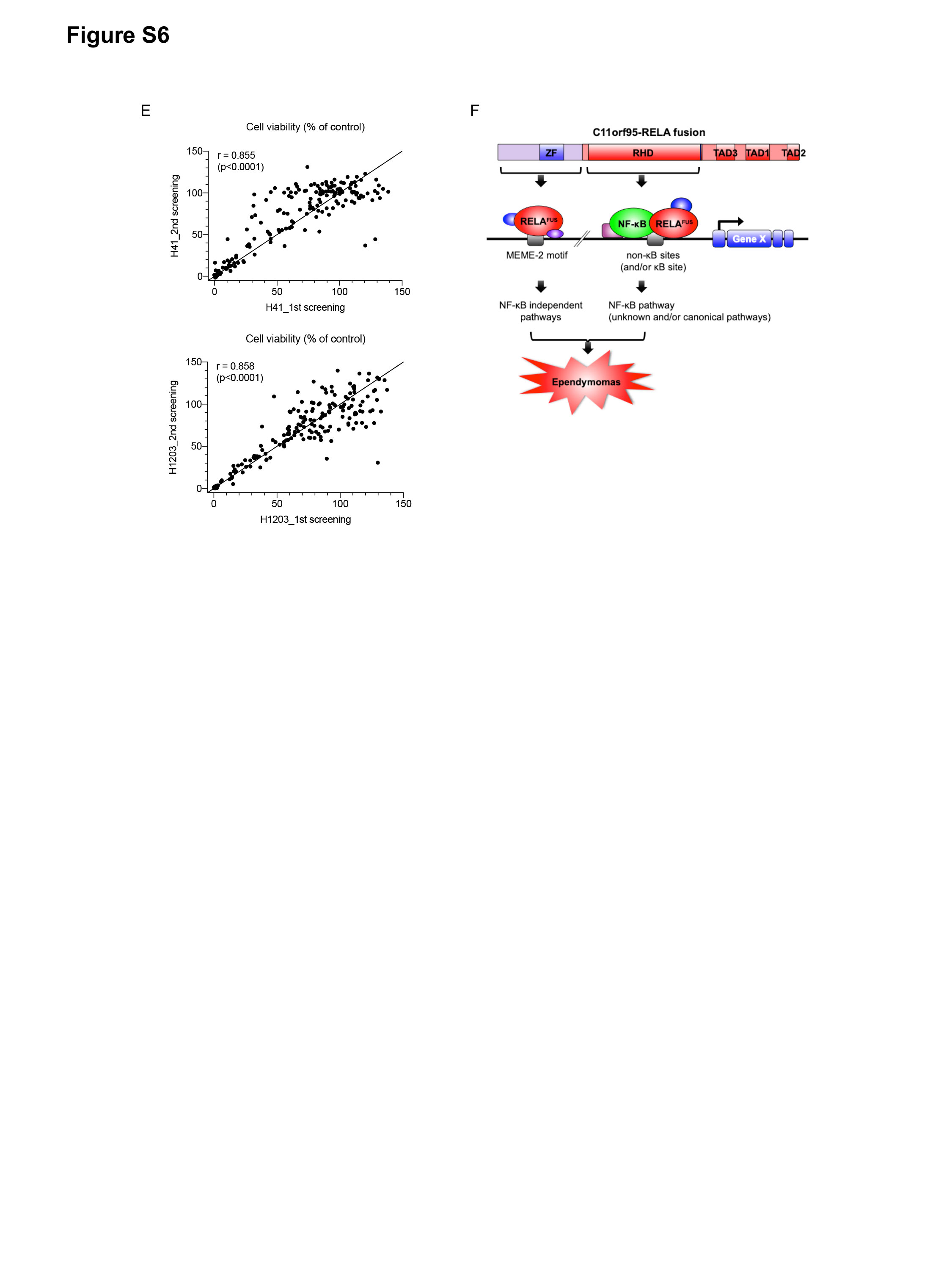

Supplement: Supplementary file 8 — Additional file8 (JPG 217 KB) [file 40478_2021_1135_MOESM8_ESM.jpg]
